# Semiautomated Electrochemical Melting Curve Analysis Device for the Detection of an Osteoporosis Associated Single Nucleotide Polymorphism in Blood

**DOI:** 10.1021/acs.analchem.3c01668

**Published:** 2023-09-15

**Authors:** Cansu Pinar Yenice, Nassif Chahin, Miriam Jauset-Rubio, Matthew Hall, Phil Biggs, Hans-Peter Dimai, Barbara Obermayer-Pietsch, Mayreli Ortiz, Ciara K. O’Sullivan

**Affiliations:** † INTERFIBIO Research Group, Departament d’Enginyeria Química, Universitat Rovira i Virgili, 43007 Tarragona, Spain; ‡ Labman Automation Ltd., Seamer Hill, Stokesley, North Yorkshire TS9 5NQ, U.K.; § Division of Endocrinology and Diabetology, Department of Internal Medicine, Medical University of Graz, 8010 Graz, Austria; ∥ Institució Catalana de Recerca i Estudis Avancats (ICREA), 08010 Barcelona, Spain

## Abstract

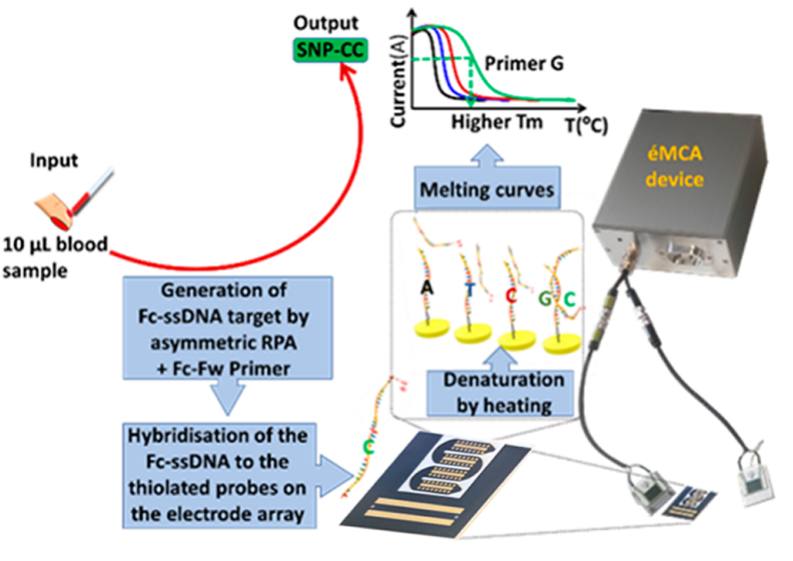

The detection of single nucleotide polymorphisms (SNPs) is of increasing importance in many areas including clinical diagnostics, patient stratification for pharmacogenomics, and advanced forensic analysis. In the work reported, we apply a semiautomated system for solid-phase electrochemical melting curve analysis (éMCA) for the identification of the allele present at a specific SNP site associated with an increased risk of bone fracture and predisposition to osteoporosis. Asymmetric isothermal recombinase polymerase amplification using ferrocene labeled forward primers was employed to generate single stranded redox labeled amplicons. In a first approach to demonstrate the proof of concept of combining asymmetric RPA with solid-phase éMCA, a simplified system housing a multielectrode array within a polymeric microsystem, sandwiched between two aluminum plates of a heater device, was used. Sample manipulation through the microfluidic channel was controlled by a syringe pump, and an external Ag/AgCl reference electrode was employed. Individual electrodes of the array were functionalized with four different oligonucleotide probes, each probe equivalent in design with the exception of the middle nucleotide. The isothermally generated amplicons were allowed to hybridize to the surface-tethered probes and subsequently subjected to a controlled temperature ramp, and the melting of the duplex was monitored electrochemically. A clear difference between the fully complementary and a single mismatch was observed. Having demonstrated the proof-of-concept, a device for automated éMCA with increased flexibility to house diverse electrode arrays with internal quasi-gold reference electrodes, higher resolution, and broader melting temperature range was developed and exploited for the detection of SNP hetero/homozygosity. Using the optimized conditions, the system was applied to the identification of the allele present at an osteoporosis associated SNP site, rs2741856, in 10 real fingerprick/venous blood samples, with results validated using Sanger sequencing.

## Introduction

Single nucleotide polymorphisms (SNPs) can be defined as the presence of alternative bases at a particular allele in a DNA sequence and occur at about one per 100–300 bp in the human genome.^1,2^ The identification of SNPs is of considerable importance for association studies of complex diseases, pharmacogenetics, patient stratification, and advanced forensics. To date, many genetic diseases have been associated with specific SNPs including the inherited forms of cardiomyopathy, cystic fibrosis,^3^ thalassemia,^4^ sickle cell anemia,^5^ retinitis pigmentosa,^6^ and osteoporosis.^7^

A plethora of genotyping technologies for the identification of SNPs via allele-discrimination have been developed, including ligation,^8,9^ enzymatic cleavage,^10^ and allele-specific hybridization.^11^ Several examples of the use of highly elegant approaches for the allele-specific hybridization and multiplexed detection of RPA products have been reported (Table 1). As can be seen in Table 1, the platforms are capable of an excellent level of parallelized multiplexing, with impressive detection limits, with detection mainly based on optical transduction. However, these techniques can be costly and complex, often requiring considerable infrastructure and instrumentation and experienced personnel with considerable hands-on time due to the multiple steps involved in the analysis.

**Table 1:** Examples of Allele-Specific Hybridization of RPA Products

analytical platform	detection method	LOD	multiplexing	detection equipment	target	ref
Colorimetric detection on chip surface	2 steps: RPA followed by allele-specific hybridization chain reaction (AS-HCR)	100 fM or 0.2% (expressed in percentage of mutated DNA with respect to total DNA (%) or concentration (fg·μL^–1^/aM)	12 targets	Smartphone	p.GirC mutation (c.34G>T) in the KRAS gene and the p.Q61K mutation (c.131C>A) in the NRAS gene	(12)
Colorimetric detection on chip surface	2 steps: blocked-RPA and allele-selective hybridization on dendron-mediated chips	0.02 ng nL^–1^ genomic DNA	7 targets	Chip reader (office scanner)	H1047R mutation in the PIK3CA gene	(13)
Colorimetric detection on lateral flow assay	2 steps: recombinase polymerase amplification coupled with lateral flow dipstick (RPA-LFD	Not detailed	Not detailed	Visual analysis	SNP F1534C	(14)
Fluorescence and colorimetric detection on DNA chip	2 steps: blocked PCR or superselective primer-PCR followed by hybridization on DNA chips for optical reading	Not detailed	12 targets	Microarray scanner or document scanner, smartphone	Single-nucleotide variations in KRAS gene at exon12 (KRAS c.34G>T, KRAS c.35G>A, KRAS c.33G>A)	(15)
Fluorescence detection on microfluidic chip	2 steps: blocked RPA followed by on-bead allele-specific hyridization	250 copies of genomic DNA	8 targets	Fluorescence reader	Substitutions of the KRAS gene at codon 12 (p.G12C (c.34G>T, rs121913530), p.G12S (c.34G>A, rs121913530), and p.G12V (c.35G>T, rs121913529)	(16)
Colorimetric detection on chip surface	3 steps: allele-specific ligation followed by RPA and hybridization on chip	Not detailed	36 targets	Microplate reader	rs1057910, rs1799853, and rs9923231 located in genes CYP2C9 and VKORC1	(17)

Hybridization based melting curve analysis approaches for SNP detection exploit the thermal stability of double stranded DNA, which is controlled by the specific sequence as well as by the presence of mismatched bases.^18^ These approaches routinely use a resistive heater to increase the temperature in a highly controlled ramp, resulting in the denaturation (melting) of the duplex, which is normally monitored via the decrease in fluorescence of intercalating dyes such as SYBR Green.^19^ The intrinsic limitations of fluorescence-based devices for multiplexed detection are related to the complexity of the optical setup, with CCD cameras typically employed for multiplexed detection requiring relatively expensive cooling units. A recent significant advancement in the multiplexed detection of SNPs was the Hydra 1K, which uses solid-phase melting curve analysis and is able to perform more than 1000 parallelized melts on a CMOS array and takes advantage of multiple integrated biosensors, for integrated PCR amplification, followed by hybridization and detection in a closed-tube system, avoiding the requirement for an external light detector.^20,21^

Recently, we have focused on the development of alternative approaches for the electrochemical determination of SNPs, including the use of redox labeled dideoxynucleotides (ddNTPs) and solid-phase single base extension.^22−24^ The use of electroanalysis for the monitoring of duplex denaturation has been reported previously using electroactive intercalators such as organometallic compounds like cobalt phenanthroline,^25,26^ cobalt bipyridine,^27,28^ ruthenium bipyridine,^29^ and osmium bipyridyl complex^30^ or organic molecules, like methylene blue (MB), echinomycin,^31−33^ and epirubicin.^34^ Alternatively, duplexes can be labeled with redox labels such as methylene blue^35,36^ and ferrocene^37,38^ during amplification using a labeled forward primer in an asymmetric PCR^21,38,39^ and subsequently detected using voltammetry.^37,38^

Recently, we developed a laboratory setup capable of simultaneous electrochemical melting curve analysis (éMCA) for the detection of a single nucleotide polymorphism.^38^ In the work reported herein, we wanted to extend on our previous work to move closer toward a true point-of-care (POC) device that could be used to identify the allele present at a specific SNP site via solid-phase melting curve analysis. To meet the requirements of a POC device, PCR thermal cycling amplification was replaced with isothermal recombinant polymerase amplification, and the use of exonuclease digestion to generate single stranded DNA was avoided, as the isothermal asymmetric amplification was highly efficient. The system was applied to the identification of the allele present at an osteoporosis associated rs2741856 SNP in real blood samples, with results validated using Sanger sequencing as previously described.^40^

Osteoporosis is a bone disease that affects around 200 million people globally.^41^ It is characterized by low bone mass and microarchitecture deterioration, resulting in fragile bones and thus increasing the probability of fracture.^42,43^ Diagnosis of osteoporosis is typically achieved by measuring the bone density by dual-energy X-ray absorptiometry and monitoring bone density at periodic intervals over a 2–3 year time frame.^44^ Recent reports have identified SNPs that are associated with an increased risk of bone fracture and can be used as markers of an increased risk of bone-fracture and as markers of a predisposition to osteoporosis.^45−48^

We thus applied our éMCA to the identification of the alleles present at the rs2741856 SNP site using solid-phase melting curve analysis. In diploid organisms like humans, two sets of homologous chromosomes share the same loci (one allele from the mother and another from the father), and the organism can be homozygous if both alleles are the same (for SNP rs2741856: GG or CC) or heterozygous at that locus if the alleles are different (GC). In individuals without any predisposition to osteoporosis, the allele at the SNP is CC, while for those with a possible predisposition, the allele at the SNP is GG or CG/GC, with an increased association with the GG allele.

**Figure 1:**
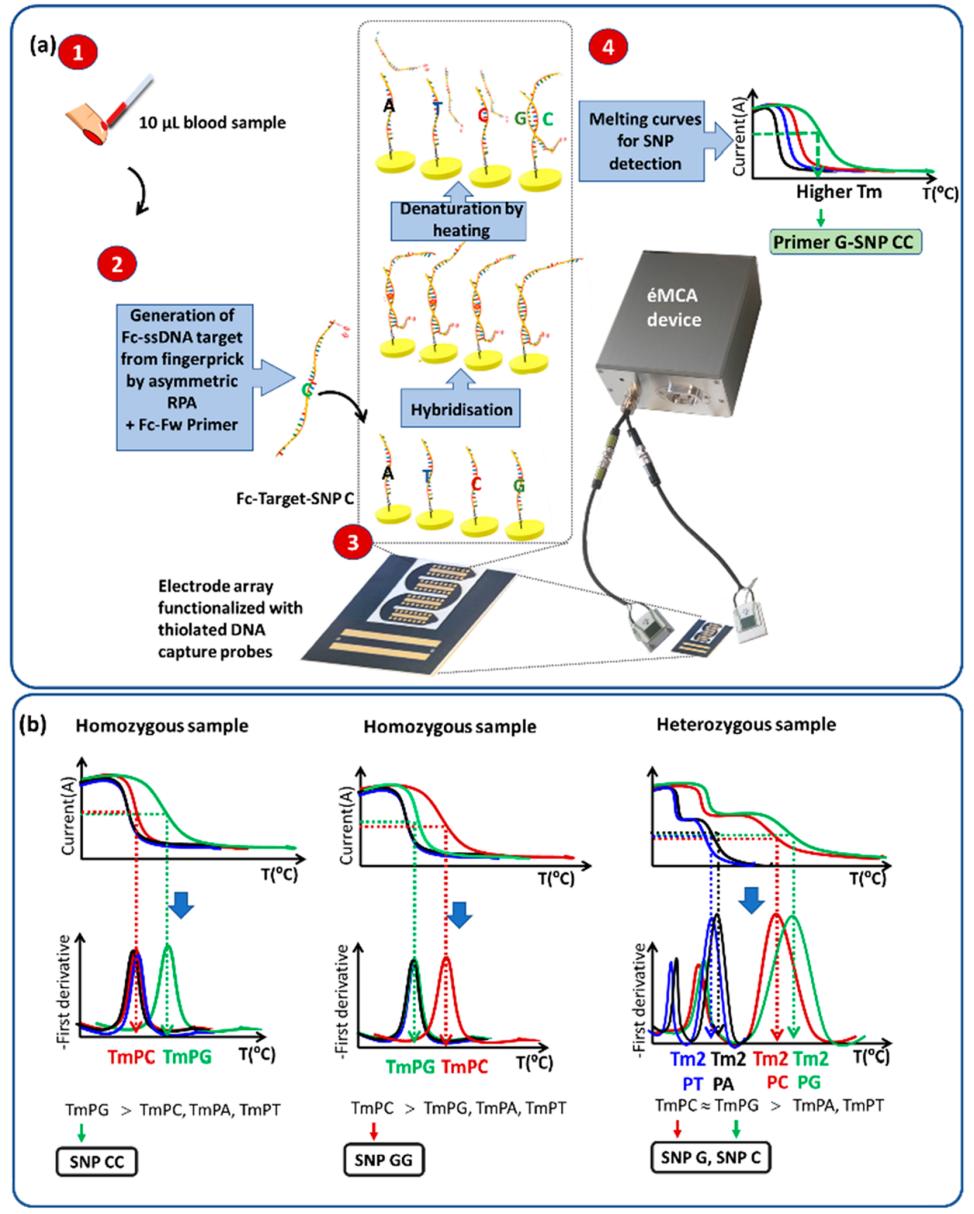
(a) Schematic representation of the approach for electrochemical melting-curve analysis: (1) a 10 μL blood sample is taken; (2) the ferrocene-ssDNA generated (Fc-Target-SNPC) using asymmetric-RPA and a Fc-labeled forward primer (Fc-Fw primer); (3) the Fc-target hybridizes to thiolated probes immobilized on individual electrodes of the array, and the resulting duplexes are subjected to thermal denaturation; (4) the melting curves constructed with the DPV responses obtained during temperature ramping. (b) Representation of the three possibilities of zygosity for a real sample for SNP rs2741856: homozygous (GG or CC) or heterozygous (GC) at that locus if the alleles are different (GC), and analyzed using the first derivatives of melting curves.

In a first demonstration of a proof-of-concept, a simple laboratory setup was used, where an array of nine gold electrodes, produced by sputtering, were housed within microfluidics, placed between two aluminum heating plates, with an external Ag/AgCl reference electrode. The microfluidics were connected to a syringe pump (Figure S1). The heating ramp was controlled, and electrochemical measurements were taken.^38^ Four different thiolated probes were used (Table S1) and were immobilized on individual electrodes of an array (Figure 1a). Each of these probes was specifically designed so that the SNP site in the single stranded isothermally generated amplicon that is under interrogation hybridizes to the middle of the immobilized probe, as previously optimized.^38^ At this specific site, each probe contained a different nucleotide, which is specifically related to the SNP or bases that are not related to the SNP, which are used as negative controls. A 138-mer ssDNA was either synthesized or isothermally generated using asymmetric recombinase polymerase amplification with a ferrocene labeled forward primer and an unlabeled reverse primer. Following hybridization, simultaneous melting curve analysis of all 8 duplexes (each probe in duplicate) was carried out, with the temperature ramped at 1 °C/step. The DPV response of the ferrocene label was measured throughout the temperature ramp, and melting curves were generated. The melting temperatures were determined using first derivate analysis (Figure 1b).

Having demonstrated the proof-of-concept with this laboratory setup, a prototype device that housed the heating plates, a small pump, and all the necessary electronic components was produced by Labman Automation, U.K. (Figures 1a, S3, and S4 and Annex of Supporting Information). This second-generation device was designed to be flexible in the types and dimensions of electrode arrays that could be accommodated, and we used an array of 64-gold electrodes, produced by screen-printing, a cost-effective alternative to sputtering, with cleaning achieved by simple immersion. This device completely automated the melting curve analysis and was connected to a 64-channel potentiostat. Using the same approach of 4 electrodes/SNP, the system was applied to the analysis of real blood samples, and the hetero/homozygosity of these samples was determined using this second-generation device and the éMCA for the simultaneous detection of the melting curves obtained at 12 electrodes (3 electrodes/probe) of the 64-electrode array. The results obtained with the éMCA were successfully validated using Sanger sequencing as previously described,^40^ and the results obtained are summarized in Table S4.

The work thus addresses the development of a more automated, easier to use device, moving toward a portable instrument for use at the point-of-need. The use of cost-effective screen-printed electrodes that can be easily cleaned and functionalized, together with asymmetric isothermal amplification, also contributes to the realization of a device deployable to low-resource settings, where minimal end-user intervention would be required.

## Experimental Section

### First Generation Device

In the initial studies to demonstrate the proof-of-concept of combining isothermal amplification to produce Fc-ss target DNA with éMCA for analysis of a SNP in a fingerprick blood sample, we exploited a simple laboratory setup (first-generation device) previously reported by our group^38^ but incorporated a new breakout box which significantly simplified and improved detection (Figure S1b).

#### Electrode Array

The electrode array (Figure S2a) was designed to have nine circular working electrodes (1 mm^2^) and a rectangular counter electrode (4 mm^2^). It was fabricated by sputtering on 75 mm × 25 mm soda-lime glass substrate (Sigma-Aldrich, Spain) as described previously, with minor modifications^49^ (see Supporting Information).

#### “In-House” Peltier Device

The Peltier device consists of an Arduino Uno board connected with two heating aluminum plates controlled by Arduino software for data visualization. The Peltier device provides robust control and ramping of temperature. The gold electrode array functionalized with DNA probes was placed between the heating plates and housed in a poly(methyl methacrylate) (PMMA) microfluidic cell that allows liquid buffer to wash the gold surface while heating as detailed previously.^38^ Arduino UNO, IRF520, the resistances, capacitors, type K thermocouples, connectors, BI BPC10 resistors, AD595, breadboard, and 2.5 W/mK thermally conductive tape were all purchased from Farnell (Madrid, Spain). For the temperature reference system, a type K thermocouple connected to a precision thermometer Hi 93531 (Hanna instruments, Bilbao, Spain) was used. The variable DC power supply PeakTech 6006D (Telonic Instruments LTD, Berkshire, U.K.) was used to supply the temperature reading system at 5.1 V.

#### Breakout Box Fabricated for First Generation Device

To interface the 9-electrode arrays implemented on glass substrates to standard laboratory potentiostats, compact breakout boxes that provided wired connections from a Samtec HSEC8-130-01-L-DV-A edge connector to an array of 15 numbered 4 mm sockets were designed and manufactured (Labman Automation, U.K.). The electrode arrays plugged into the edge connector and the electrodes could then, in turn, be addressed by an Autolab model potentiostat/galvanostat 12 controlled with GPES software by connecting with flying leads to the relevant 4 mm sockets. A ground connection was provided to aid the reduction of electrical noise reduction.

### Second-Generation Device

Having demonstrated the proof-of-concept, a device (second-generation device) for automated melting curve analysis was developed (Figure 1 and Figures S3 and S4 and Annex of Supporting Information).

#### Electrode Array

The second-generation device was designed to be flexible with respect to the dimension and type of electrode arrays that can be incorporated. A 64-electrode array (Figure S2b) was designed at URV and fabricated using screen-printing technology by C-MAC (Belgium). The electrodes and electrical contacts were printed with gold ink, while the tracks were printed using silver ink on one side of a 635 μm thick ceramic substrate. The final dimensions of each panel were 54.93 mm × 55.89 mm. The array contains 64 gold working electrodes (1.0 mm diameter and 8–10 μm gold thickness) having common gold counter and gold pseudoreference electrode, and the conductive tracks of the electrode array were insulated with a solder mask.

Arduino UNO, IRF520, the resistances, capacitors, type K thermocouples, connectors, BI BPC10 resistors, AD595, breadboard, and the 2.5 W/mK thermally conductive tapes were all purchased from Farnell (Madrid, Spain), while 5v DC small pump code 702-6894 washing was purchased from RS PRO. The variable DC power supply PeakTech 6006D (Telonic instruments LTD, Berkshire, U.K.) was used to supply the temperature reading system at 5.1 V. New software was programmed using Microsoft Visual Studio with a GUI interface to control the whole éMCA process besides the GPS software for a fully automated process. Two aluminum blocks were manufactured at Labman. The top block contained two holes for the inlet and outlet. Small steel bar fittings were used to attach the tubes. The aluminum top block replaced the previous Perspex block, which had been used to verify the liquid flow through the microfluidic channel. The Arduino Uno original script was modified to integrate the Arduino heater and syringe pump (Figures S3–S6).

#### Breakout Box Fabrication for Second Generation Device

This “breakout” box (Figure 6 and Figure S4) allows the connection of the common reference and counter electrodes and each individual working electrode to the potentiostat. The electrode arrays were designed to mate with a Samtec FSI- 140-03-G-D-AD one-piece interface in order to connect to external systems.

### Asymmetric RPA Reaction

The ferrocene labeled single-stranded DNA (Fc-ssDNA) containing the osteoporosis associated SNP-site was generated using asymmetric isothermal recombinase polymerase amplification (asymmetric-RPA), where the forward primer was labeled with ferrocene at the 5′-end (Fc-FwP, Table S2) and was used at a higher concentration than the nonmodified reverse primer. The PCR-generated dsDNA target (final concentration in the mixture = 100 pM) or the genomic DNA extracted from blood sample was added to the RPA mixture and the temperature was kept constant at 37 °C for 15 min. The master mix contains the 2× reaction buffer, 10× basic E-mix buffer, and 20× core reaction mix at 1× final concentration, 2 mM dNTPs, 14 mM Mg(OAc)_2_, 5000 nM ferrocene labeled forward primer, and 200 nM reverse primer. A nontemplate control (without target) was also produced to evaluate the approach.

The RPA products were visualized using 2.6% w/v agarose gel Tris-borate-EDTA buffer as described in the Supporting Information. An oligo Clean & Concentrator kit was used to purify the products for electrochemical measurements. Optimization of the assay time and the primer ratio optimization were performed to obtain the desired conditions (see Supporting Information). Finally, the use of exonuclease digestion in addition to asymmetric RPA was also explored. For this reaction, a phosphorylated reverse primer (Table S2) was used instead of the natural primer in the RPA mixture, which is needed for the digestion reaction of lambda exonuclease.^38,50^

Blood samples (10 μL) were added to 40 μL of 5 mM Na_2_EDTA solution prepared with DNase free water, which was then heated at 95 °C for 30 s and finally left to cool to room temperature (∼22 °C).^50^ Sample 1 was a fingerprick blood sample, tested on both devices, and the other samples were Biobank blood samples obtained in a previous study.^40^ The treated blood sample was mixed with 100 μL of asymmetric-RPA master mix to generate the Fc-ssDNA target.

### Electrochemical Measurements and Melting Profiles

The working electrode array was housed within the microfluidics, and 15 μL of Fc-labeled asymmetric-RPA product in 10 mM Tris buffer (pH 7.4 at 25 °C) containing 0.5 M NaCl was injected into the channel to allow the hybridization. Following a 20 min incubation at 22 °C, the sensor was washed 3 times and placed between the heating aluminum plates of the homemade resistive heater, and the temperature increased at a rate of 1 °C/step. Differential pulse voltammograms (DPV) of the ferrocene redox label were recorded after the sensor had been exposed to each temperature starting from 25 to 40 °C. The parameters employed in the DPV measurements were for the first generation device using a potential window between 0 and 0.6 V (vs Ag/AgCl 3 M KCl) and for the second-generation device using a potential window between 0 and 0.3 V (vs Au pseudoreference) included in the array, step potential 10 mV, modulation amplitude 10 mV, modulation time 0.015 s, and interval time 0.1 s. Every melting curve analysis was carried out in duplicate, and the remaining electrode was modified with the backfiller to evaluate the nonspecific binding and in 10 mM Tris buffer (pH 7.4). The melting profiles were constructed by plotting the peak current of each DPV signal vs temperature. Results obtained to demonstrate the proof-of-concept (Figures 4 and 5) were obtained using the 9-electrode array and the simple laboratory setup (Figure S1), and the error bars represent the average and RSD% of 2 measurements/probe. The results obtained to determine the hetero/homozygosity of the interrogated SNP in real blood samples were obtained using a 64-electrode array, housed within the automated device (Figure S3), and the error bars represent the average and RSD% of 3 measurements/probe. In both cases, the biphasic dose response function was applied for fitting the curves using the Levenberg–Marquardt iteration algorithm, and finally, the melting temperature was obtained from the extrema of the first derivative.

## Results and Discussion

In previous work,^38^ PCR amplification and exonuclease digestion were employed to generate ferrocene labeled single stranded DNA from a sample. To move closer to implementation of the assay at the point-of-care/need, this thermal cycling and exonuclease digestion was replaced with isothermal asymmetric RPA, where the reaction takes place at 37 °C with amplification time reduced to just 15 min.

During developmental work, after amplification, gel electrophoresis was used to visualize the single stranded DNA amplicon and electrochemistry was used to detect the electroactive ferrocene label. The initial electrochemical trials were carried out in a simple laboratory setup (first-generation device) and using PCR-generated dsDNA as target to mimic the genomic DNA.

### Optimization of Asymmetric RPA for Fc-ssDNA Generation

**Figure 2:**
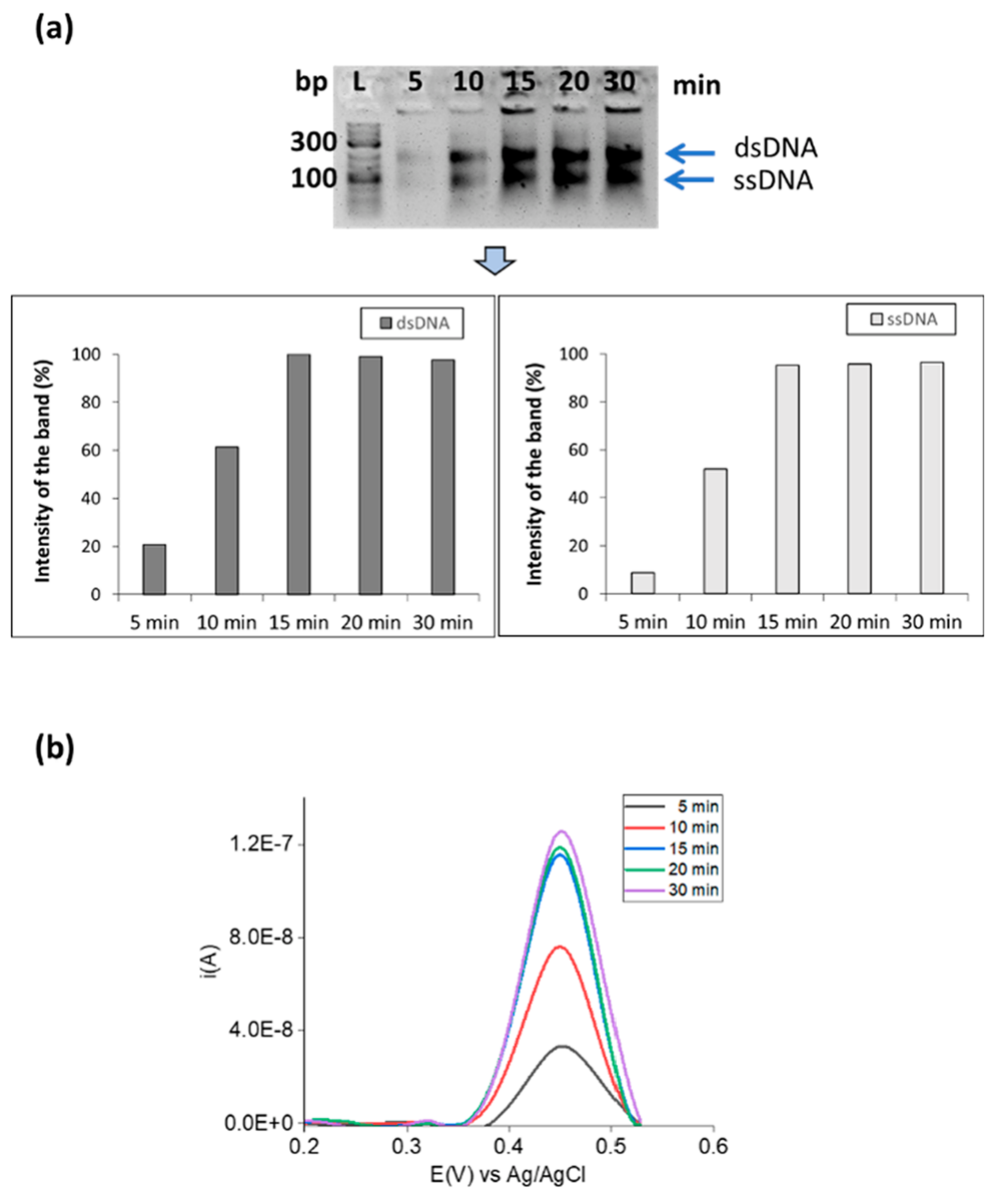
Optimization of assay time: (a) 2.6% w/v agarose gel after electrophoresis of asymmetric-RPA products obtained using synthetic dsDNA and different amplification times (5, 10, 15, 20, and 30 min) and the graph obtained using ImageJ software to calculate the intensity of the band. The values were normalized using the highest intensity values obtained in 30 min as reference for the dsDNA and the ssDNA respectively. (b) DPV responses after hybridization of asymmetric-RPA products obtained using synthetic dsDNA and different amplification times (5, 10, 15, 20, and 30 min) to surface immobilized probes. Current (amperes) = *i* (A). Duplicates were carried out for each experiment.

The amplification time needed to obtain the maximum amount of single stranded DNA was determined by carrying out the RPA reaction at different times from 5 to 30 min from a common master mix containing the RPA reagents, dNTPs, and a 5× excess of Fc-FwP with respect to the Rev-Primer and a double stranded DNA as target (Table S2 for DNA sequences). The gel electrophoresis (Figure 2a) clearly shows the bands corresponding to the double and single stranded DNA. The bands increase in intensity from 5 to 15 min and remain constant after this time.

The presence of ferrocene allows the direct evaluation of the amount of generated single stranded DNA by recording the electrochemical ferrocene signal after the hybridization of RPA products to a complementary capture probe. In agreement with the gel electrophoresis, Figure 2b also shows an increase of the electrochemical signal from 5 to 15 min, after which there is no further increase in the signal. Fifteen-minute amplification was thus chosen for all further experiments.

**Figure 3:**
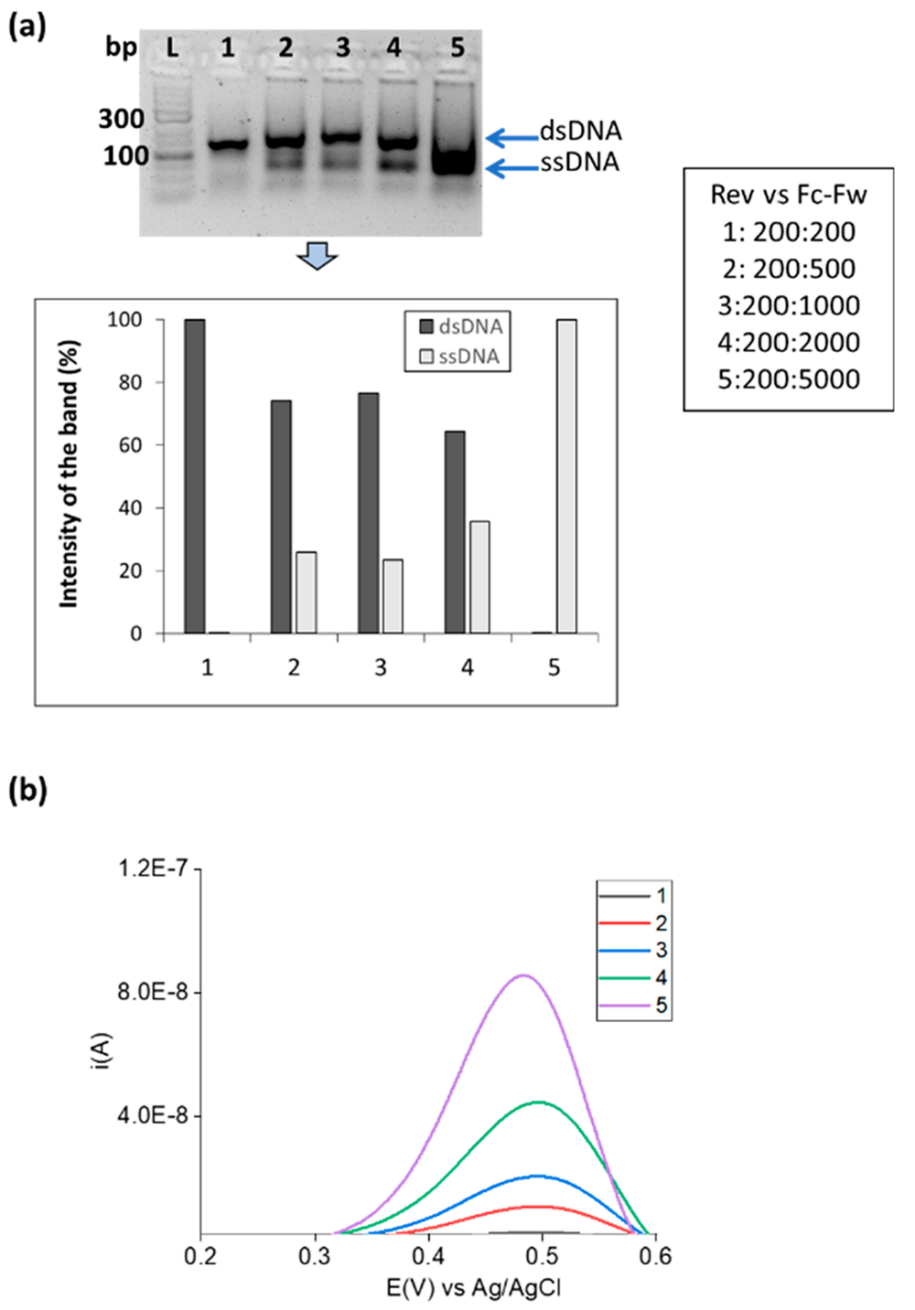
Primer concentrations optimization: (a) 2.6% w/v agarose gel after electrophoresis of asymmetric-RPA products obtained using synthetic dsDNA and different primer concentrations and the graph obtained using ImageJ software to calculate the intensity of the band. The values were normalized using as reference the sum of the values obtained for the dsDNA and ssDNA for each well separately and divided for the amount of dsDNA or/and ssDNA separately. Ratios of Fc-FwP:RevP in nM are (1) 200:200, (2) 500:200, (3) 1000:200, (4) 2000:200, (5) 5000:200, and L= ladder. (b) DPV recorded in 10 mM Tris buffer (pH 7.4) after hybridization of asymmetric-RPA products obtained using synthetic dsDNA and different primer concentrations to an immobilized probe. Current (amperes) = *i* (A). Duplicates were performed for each experiment.

Different primer ratios (Table S3) varying from 1:1 Fc-Fw Per:RevP ratios (200:200 nM) to 25:1 (5000:200 nM)) were then evaluated. As can be seen in Figure 3a, the optimum concentration of Fc-FwP to obtain a maximum amount of ferrocene labeled ssDNA was 5000 nM, which represents a ratio of 25:1 with respect to the 200 nM reverse primer concentration. Using this concentration ratio, no dsDNA is observed in the gel, indicative that all the DNA is single stranded. A higher excess of Fc-Fw did not improve the yield and purity of the ferrocene labeled product (Figure S7).

It should be noted that we also tested the use of phosphate labeled reverse primer and lambda exonuclease digestion following asymmetric RPA, but no improvement in band intensity was obtained (Figure S8) since the amplified product is in Fc-ssDNA form. For further experiments this exonuclease digestion step was omitted and a 25:1 molar ratio of forward to reverse primer employed. This improved efficiency of asymmetric amplification achieved using RPA, combined with the ability to carry out amplification at 37 °C for just 15 min, highlights the compatibility of this approach for potential future implementation at the point-of-need and considerably reduces the time and hands-on effort required to prepare the ssDNA.

Figure 3b also supports that (5000 nM:200 nM/25:1) Fc-FwP:RevP is the optimum concentration ratio since all the dsDNA is converted to ssDNA, with a considerably higher electrochemical signal obtained as compared with the other formulations.

Using these optimized conditions (15 min, 37 °C, and 25:1 Fc-FwP:RevP), the device was then applied to the identification of a specific SNP associated with osteoporosis. A Fc-ssDNA product was generated by using asymmetric RPA and a 138-mer synthetic dsDNA template. Based on our previous work,^38^ where the optimum position on the immobilized probe for hybridization to the SNP site under interrogation was demonstrated to be in the middle, four probes were designed with each containing a different base (A, G, T, or C) at this position, which are defined respectively as probe A, probe T, probe C, and probe G (Table S1). Probe C is fully complementary to the most representative allele found in the majority of the population (SNP G), and in consequence a synthetic 138-mer DNA was designed with the presence of this SNP. The other allele that can be present, but with lower frequency, is SNP C; thus probe G can also be found. Probes A and T are nonrelated to the SNP and were considered as negative controls. The electrode arrays were functionalized with each of these probes (in duplicate) with the ninth electrode of the array serving as a control to evaluate any nonspecific binding events. These functionalized electrode arrays were housed within microfluidics and then placed between the two aluminum plates of the in-house heating device (Figure S1). The ssDNA RPA amplicons were injected into the microfluidics, and hybridization with the immobilized probes was allowed to take place for 20 min at room temperature (∼22 °C). Following hybridization, the microfluidics were connected via tubing to a container with wash buffer, and this washing buffer was driven through the microfluidics via syringe pump actuation. The electrode array was then subjected to simultaneous melting curve analysis using the previously optimized temperature ramp of 1 °C/step,^38^ with washing buffer flowed over the array to continuously remove the denatured and liberated Fc-labeled DNA, and the DPV signal was measured throughout the melt (Figure S9).

**Figure 4:**
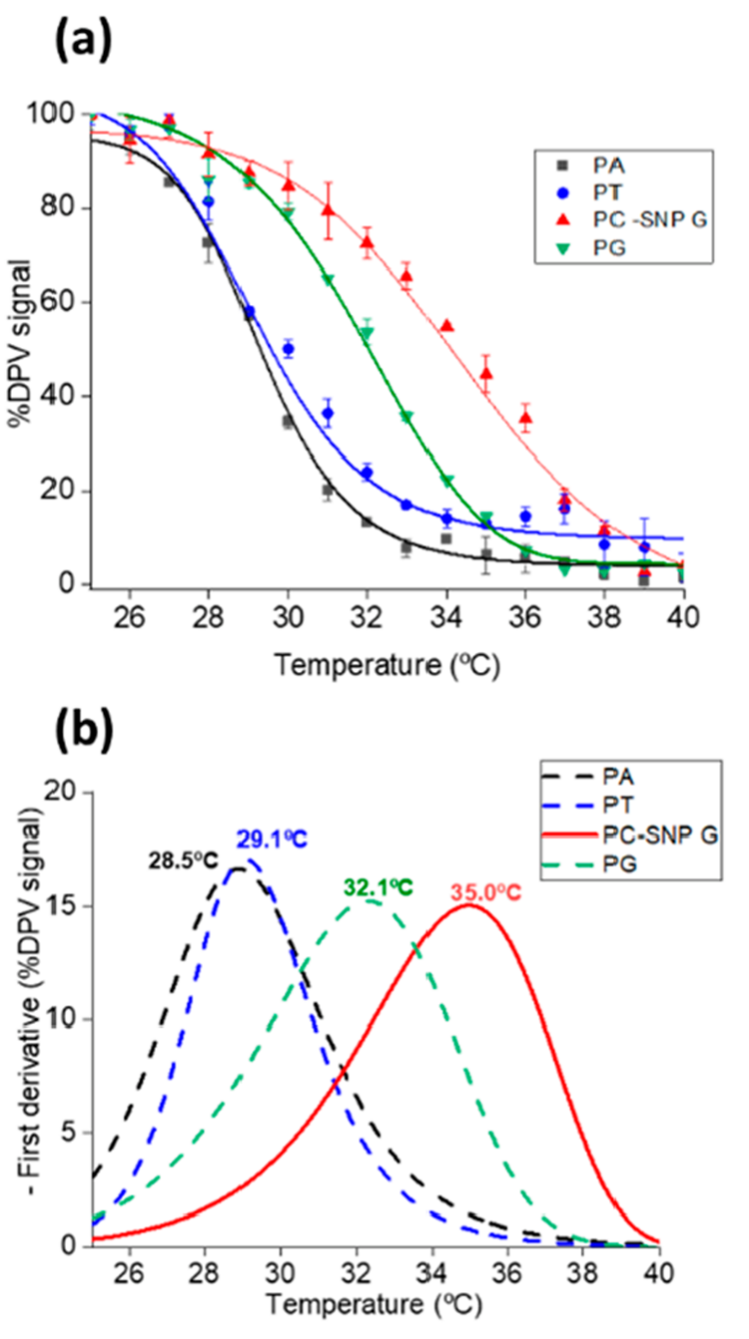
SNP detection of synthetic sample: (a) éMCA profiles generated DPV signals of ferrocene label at each with ramping (1 °C/step) using synthetic DNA as a target; (b) the corresponding first derivatives. Duplicates were performed.

The melting curves obtained using the four different probes with the same RPA generated amplicon are shown in Figure 4a and their first derivative curves in Figure 4b. The melting profiles were constructed as percentages of the DPV signal referred to the initial signal during ramping temperature (analytical data are shown in Figure S15). The melting temperatures were calculated for the capture probe PC, which was fully complementary to the target carrying SNP G and was 35.0 °C, while considerably lower melting temperatures of 32.1 °C were obtained for the other SNP related probe PG and the negative controls PA (28.5 °C) and PT (29.1 °C), respectively. This simultaneous melting curve analysis approach could thus clearly identify the allele present at the SNP site.

### SNP Detection in Real Samples

**Figure 5:**
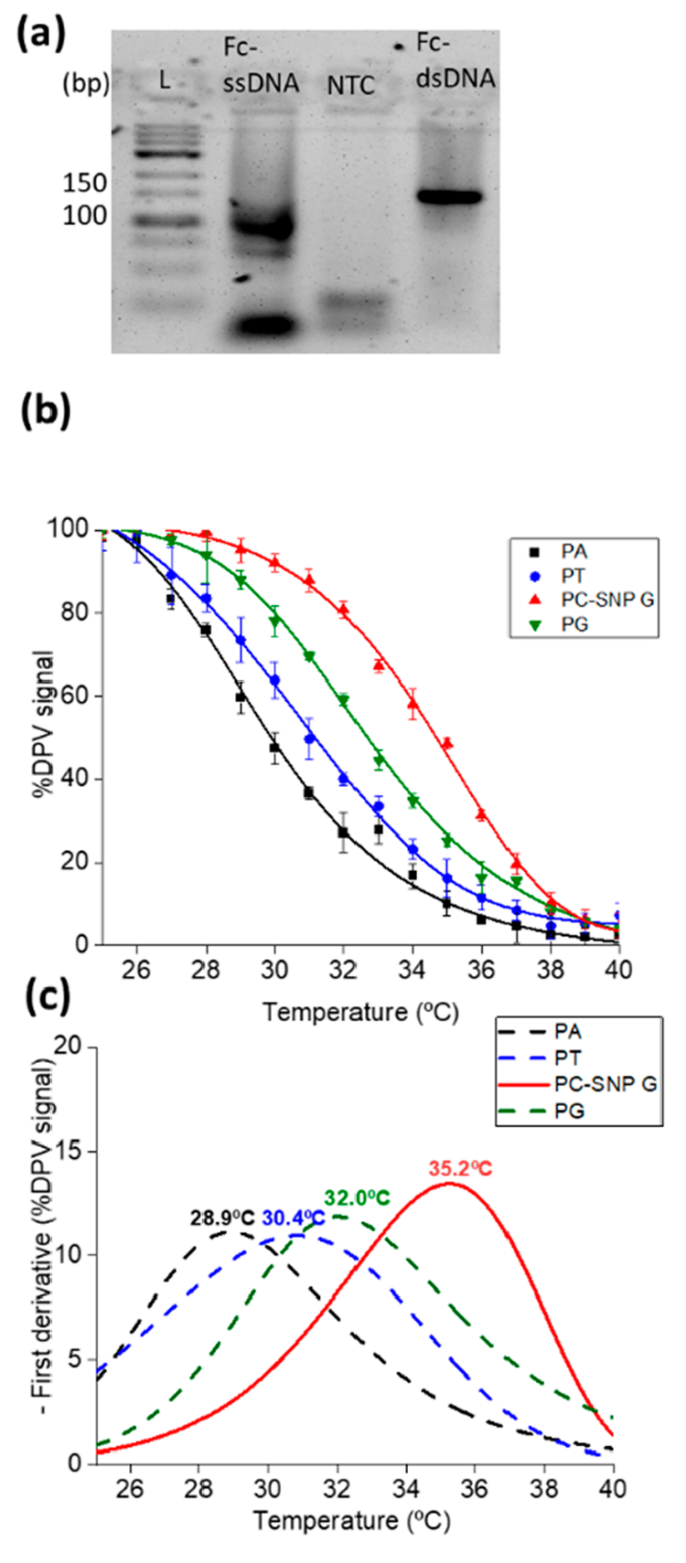
Real fingerprick blood sample (sample 1). (a) Gel electrophoresis image after Fc-ssDNA generation from real sample 1 using asymmetric-RPA (double stranded DNA was also produced using RPA in blood in a different reaction and included in the gel for comparison. (b) éMCA profiles by recording DPV with *T* ramping (1 °C/step). (c) Corresponding first derivatives. Duplicates were carried out.

Having demonstrated the proof-of-concept of using simultaneous melting curve analysis using diverse probes on individual electrodes to identify the allele found at a specific SNP site with the synthetic DNA, the device was then applied to real sample analysis using an areal blood sample. Ten microliters of blood was diluted in 5 mM Na_2_EDTA, then heated at 95 °C for 30 s and finally left to cool to room temperature (∼22 °C).^51^ The heat-treated blood sample was added to the asymmetric-RPA master mix to generate Fc-ssDNA labeled target (Figure 5a), which was then added to the functionalized electrode array and simultaneous melting curve analysis carried out as described above (Figure S10, Figure 5b,c). In this case, a similar trend was observed with the fully complementary duplex with the probe containing the C base in the middle having a melting temperature of 35.2 °C, while the probe containing the G base had a melting temperature of 32 °C, indicating that the individual of that sample is homozygote for the SNP 27, having both alleles the same SNP G. Moreover, the negative control probes PA and PT have very similar melting temperatures of 28.9 and 30.4 °C, respectively.

This device was successfully employed to detect SNPs in homozygote samples but lacks the resolution needed to detect heterozygosity. Therefore, a second generation of devices with a greater degree of automation was developed for the detection of SNPs and heterozygosity in real samples.

**Figure 6:**
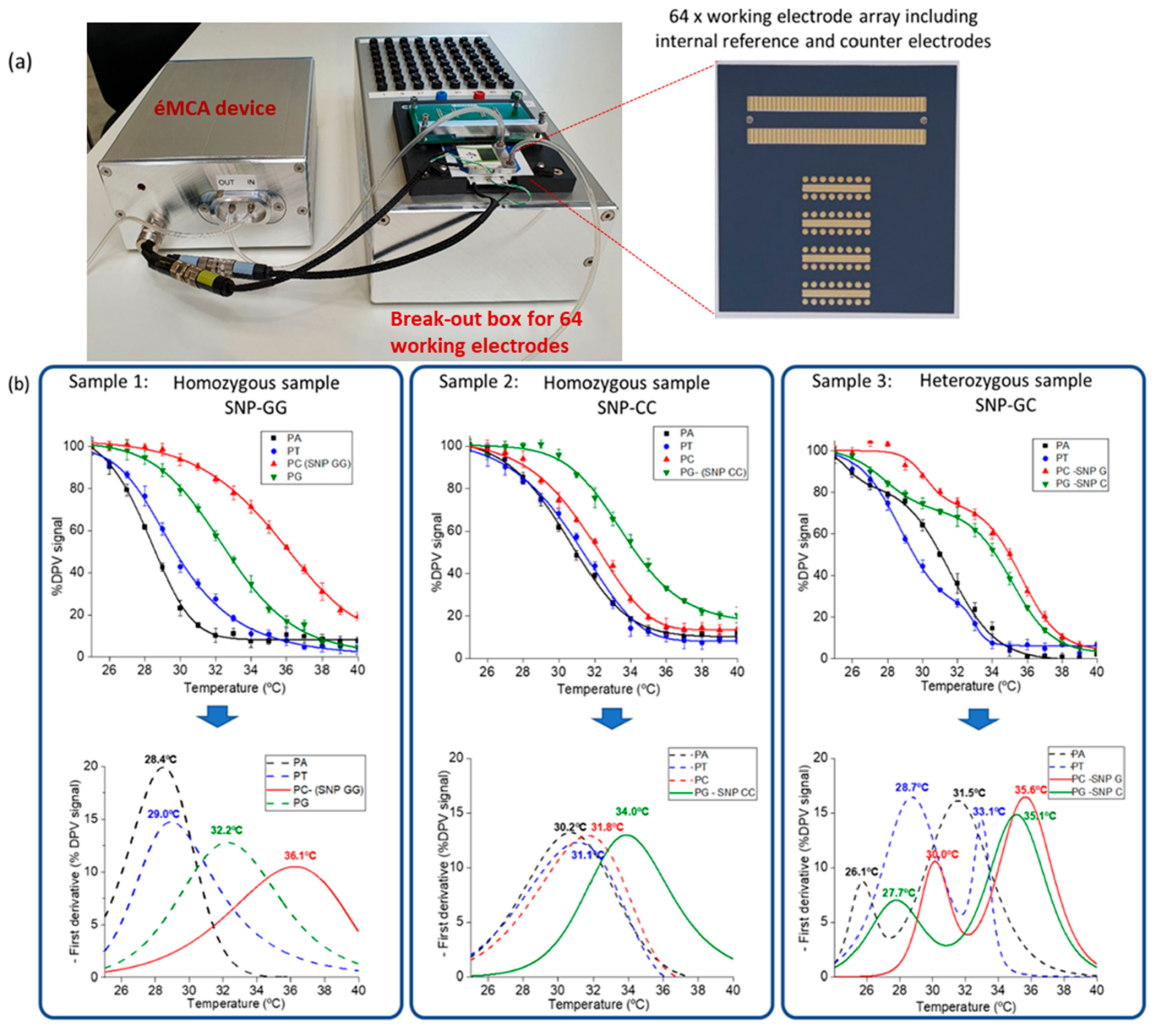
Analysis of real blood samples with the second-generation device. (a) Photo of the second-generation prototype device with the breakout box for simultaneous éMCA and the 64-electrode array screen-printed on ceramic substrate. (b) éMCA profiles obtained by recording DPV with *T* ramping (1 °C/step) of 3 representative blood samples and the corresponding first derivatives. Triplicates were carried out for each probe and sample used.

This more compact setup for éMCA is shown in Figures 1 and 6. This new setup is composed of two parts (Figures S3 and S4), the heating-device box which contains the Arduino board, a small pump, and the breakout box where the 64-electrode array is connected to the multichannel potentiostat. The ceramic screen-printed electrode array is placed between the top and bottom heating blocks, where the top block is adhered to the array surface using a double-sided adhesive gasket to cover all of the electrodes, with the top block containing two holes at the corners for introduction of the liquid from the pump to the electrode array. The serpentine configuration for the microfluidics (Figures 1 and S3) facilitates sealing of the chamber and maintains the liquid moving smoothly, avoiding the introduction of air bubbles that can interfere with the electrochemical measurement. This serpentine configuration also allows all of the electrodes to be covered at the same time, increasing the reproducibility of the response.

This second-generation prototype has several advantages over the previously used in-house produced setup. The first one is the avoidance of the use of an additional XL-Cavro syringe pump for washing the array as well as removal of the requirement of an additional laptop to control the Arduino heating board. Additionally, the actuation of the washing syringe is completely automated and the data automatically stored throughout the entire temperature ramping process (Figures S4 and S6). Finally, the use of a 64-electrode array (the maximum number of electrodes defined by the maximum number of channels in the Autolab multichannel potentiostat) increases the number of SNPs that can be detected simultaneously, and the use of internal quasi-reference gold electrodes also further simplifies the setup, avoiding the use of an external reference electrode. While in the work reported here we analyzed one specific SNP, the same electrode array could easily be extended to simultaneously analyze 16–32 SNPs (dependent on the negative controls used).

For comparison with the first-generation device, the same fingerprick blood sample was analyzed by the second-generation device (Figure 6b and Figure S11 sample 1). The expected decrease of the DPV signal with temperature ramping was observed. The possibility of carrying out the measurements in triplicate increased the intraarray reproducibility as can be seen in Figures 6b and S12. In addition, the protective insulating layer that surrounds the working electrode in the 64-electrode array supports the drop during spotting and incubation of the primers. In the case of the 9-electrode configuration array, this insulating layer is not present and additionally these electrodes had a slightly rougher surface than the 64-electrode array resulting in higher signals and a lower reproducibility (Figure S12) for electrodes functionalized with the same primer and hybridized with the fully complementary Fc-ssDNA (SNP G). The interarray reproducibility was also observed to be better for the 64-electrode configuration (Figure S13).

As observed in Figure 6, the accuracy of the new device allows the analysis of the heterozygosity/homozygosity of the samples (sample 3) and also opens the possibility of the analysis of several SNPs in the same sample. The method is sensitive enough to differentiate between sequences with one mismatch which is equivalent to a difference in Tm as low as 1 °C.

Both a fingerprick blood sample (sample 1) and further blood samples provided by the Medical University of Graz Biobank in a previous study^40^ were analyzed following asymmetric RPA (Figure S14), and the éMCA results are shown in Figure S15 (for the raw data) and Figure S16 (including first derivatives). The rationale for using Biobank blood samples was to have samples representing both homo- and heterozygosity, and they were used to demonstrate the ability of the approach to be able to detect and differentiate between homozygosity and heterozygosity.

As observed in Figure 6, the first derivative plots clearly show only one peak for samples 1 and 2, indicating that the individuals are homozygote (sample 1-GG and sample 2-CC), which is also the case of samples 4, 5, 7, 8, 9, and 10 (Figure S16), while for samples 3 (Figure 6) and 6 (Figure S16) their heterozygote nature (GC) is evidenced by the presence of two peaks in their first derivative plots. The results obtained from éMCA and the Sanger sequencing are 100% in agreement (Table S4), highlighting the robustness and reliability of the éMCA and its compatibility with fingerprick and venous blood samples.

## Conclusions

We report on the application of electrochemical melting curve analysis (éMCA) to the detection of single nucleotide polymorphism (SNP) associated with a predisposition to bone fracture and osteoporosis. Building on previous work and with the objective of moving toward a completely automated point-of-care/need device, we employed asymmetric isothermal recombinase polymerase amplification (RPA) for the generation of a ferrocene labeled single stranded amplicon (Fc-ssDNA) containing the SNP site to be interrogated. The amplification time and primer concentrations for generation of the ssDNA target associated osteoporosis rs2741856 were optimized to be 15 min and 25:1 molar ratio, Fc-FwP:RevP, respectively. In a first-generation laboratory setup, which housed arrays of nine electrodes, produced by sputtering, within microfluidics, which was then placed between two heating blocks and connect to a syringe pump, a proof-of-concept of the combination of RPA generated Fc-ssDNA and electrochemical melting curve analysis. Having demonstrated the proof-of-concept, a second-generation device, which completely automated the melting curve analysis, was developed. This device houses a small pump and heaters, is designed to be flexible in the types of electrode arrays that can be accommodated for éMCA, and is completely automated, simply requiring addition of the Fc-ssDNA amplicons. Screen-printed electrode arrays, which are cost-effective, scalable electrode array, were employed in this second-generation device, and the system was applied to the detection of the hetero/homozygosity of the osteoporosis SNP under interrogation. Real fingerprick and venous blood samples were analyzed, and the results were validated by Sanger sequencing, and a 100% correlation was obtained.

Addressing the potential true implementation of the developed device for use at the point-of-care/need, the first issue that needs to be addressed is stability. We have previously demonstrated the stability of the functionalized electrodes to be >2 years when stored at room temperature/refrigerated conditions.^52,53^ RPA reagents are available as lyophilized pellets and could thus avoid refrigerated storage; currently, these pellets can be stored at 4 °C for 2 years. Addressing portability and cost-effectiveness, the current laboratory prototype weighs 1.9 kg and is 20.4 cm (h) × 15.5 cm (w) × 8 cm (l) in dimension and has an estimated current production cost of <USD100 (parts only), and the device has been designed with scalability in mind. Labman Automation is currently finalizing the development of a compact 64-channel potentiostat that can be integrated with the device, and electrochemical detection will then also be activated and automated via the device software. This potentiostat is capable of simultaneous rather than sequential detection and has a 60 times faster measurement speed compared to typical multiplex commercial potentiostat. Addressing costs, one potential hurdle could be the RPA reagents as they are currently only available from one source, but several companies started offering RPA reagents (e.g., New England Biolabs, Intact Genomics), which should circumvent this potential hurdle. The screen-printed electrode arrays used are not only cost-effective, but due to the high-temperature curing used in their manufacture, they can be easily cleaned by simple water immersion, with the individual electrodes of the array functionalized with an automatic spotter, highly compatible with mass manufacture. Bulk purchasing of electric components, parts, and reagents and upscaling will result in reduced reagent requirement and will also contribute to a reduction in costs.

Current work is focused on the development of a third-generation device where the potentiostat will be integrated and the running software will be updated to also activate electrochemical measurements, with the final readout being the melting temperature obtained for each electrode of the 64-electrode array. A fourth-generation device could also incorporate thermal lysis and the RPA amplification with automated addition of the Fc-ssDNA(s) to the electrode array, with the only end-user intervention thus being addition of a fingerprick blood sample. In parallel, using the second-generation device, the simultaneous multiplexed detection of diverse SNPS in a single fingerprick blood sample is being pursued.

## Supporting Information Available

Reagents details; experimental details of electrode array fabrication; electrochemical cleaning of probe immobilization; sequence design; PCR generation of dsDNA; gel electrophoresis; asymmetric RPA; tables detailing target and primer sequences, primer concentrations, sequencing results; figures including photos and schematics of first and second generation devices, electrode arrays, gel electrophoresis of optimization of primer concentration for asymmetric RPA and ssDNA generation with and without exonuclease digestion; raw DPV data for melting curve analysis; comparison of performance with first and second generation devices; gel electrophoresis of ssDNA RPA products from fingerprick and blood venous samples; raw DPV data from first and second generation devices; first derivative analysis; user manual for second generation device (PDF)
