# Supplementary material for: Semiautomated Electrochemical Melting Curve Analysis Device for the Detection of an Osteoporosis Associated Single Nucleotide Polymorphism in Blood
Source: Anal Chem. 2023 Sep 15;95(38):14192–202. doi: 10.1021/acs.analchem.3c01668 (PMC10534999; doi:10.1021/acs.analchem.3c01668)
Supplement: Supplementary file 1 — ac3c01668_si_001.pdf [file ac3c01668_si_001.pdf]

SUPPORTING INFORMATION

Semi-automated electrochemical melting curve analysis device for the detection of an osteoporosis associated single nucleotide polymorphism in blood

Cansu Pinar Yenice,[1‡] Nassif Chahin, [1‡] Miriam Jauset-Rubio,[1] Matthew Hall[2], Phil Biggs,[2] Hans-Peter Dimai, [3] Barbara Obermayer-Pietsch,[3] Mayreli Ortiz, [1*] and Ciara K. O'Sullivan[1,4*]

[1] INTERFIBIO Research Group, Departament d'Enginyeria Química, Universitat Rovira i Virgili, 43007 Tarragona, Spain

[2] Labman Automation Ltd. Seamer Hill, Stokesley, North Yorkshire, TS9 5NQ, UK.

[3] Division of Endocrinology and Diabetology, Department of Internal Medicine, Medical University of Graz, Graz, Austria

[4] Institució Catalana de Recerca i Estudis Avancats (ICREA), 08010 Barcelona, Spain

Corresponding authors: **Email:** ciara.osullivan@urv.cat ; mayreli.ortiz@urv.cat

Index

Experimental Section..4

Sequences design. 5

Preparation of the dsDNA from synthetic 138-mer ssDNA by PCR, for further RPA amplification...5

Gel electrophoresis Analysis. 5

Assymmetric RPA reaction. 5

Tables 6

Table S1. Sequences of target and capture probes related with SNP used for éMCA (each one has a SNP in the middle). The regions of the target sequence complementary to the capture probes are underlined) and also the fully complementary capture probe: Probe-C. SNP-GG related to osteoporosis..6

Table S2. Primers for dsDNA generation by PCR and for RPA reaction 6

Table S3. Ratio between forward and reverse primers used for optimisation of the asymmetric-RPA..7

Table S4. Correlation between the SNP detected by the éMCA method and Sanger sequencing (used as reference method). 7

Figures..8

Figure S1. (a) First generation of the set-up; (b) Break-out box developed by Labman for this project. 8

Figure S2. Electrode arrays used in the present work (a) 9 x gold working electrode array and (b) 64 x gold working array. 8

Figure S3. Real picture of the box containing a small pump and Arduino board with the internal view in the inset. The assembly of the 64 x working electrode array for the melting procedure for the second generation device. 9

Figure S4. (a) Top and front views of the second generation of the set-up developed for the present work.(b) Electrochemical Melting Curve System diagram. (c) Heater and channel detail. 10

Figure S5. Screenshot of the software specifically developed to run the electrochemical melting curve analysis prototype showing how the flow rate of the washing buffer and the rate of the temperature ramps are controlled, and the temperature continuously measured throughout. 11

Figure S6. Screenshot of the continuous DPV measurement throughout the temperature ramp. 11

Figure S7. Preparation of ssDNA with a higher excess of Fc-Forward primer than the used in Figure 3 of the manuscript (2.6 % w/v agarose gel after electrophoresis of asymmetric-RPA products obtained using PCR generated dsDNA as target) 11

Figure S8. Preparation of ssDNA with and without lambda exonuclease digestion: (a) 2.6 % w/v agarose gel after electrophoresis of asymmetric-RPA products obtained using PCR generated dsDNA as target and with and without the addition of lambda exonuclease; (b) the graph obtained using Image J software to calculate the intensity of the band. 12

Figure S9. Differential pulse voltammograms (DPV) recorded for each of the four probes hybridised to the Fc-labelled synthetic target after the sensor had been exposed to each temperature starting from 25 until 40 °C. The parameters employed in the DPV experiments were: potential window between 0 and 0.6 V (vs. Ag/AgCl 3M KCl), step potential 10 mV, modulation amplitude 10 mV, modulation time 0.015 s and interval time 0.1 s. 12

Figure S10. Differential pulse voltammograms (DPV) recorded for each of the four probes hybridised to the Fc-labelled ssDNA generated from blood (Sample 1) after the sensor had been exposed to each temperature starting from 25 until 40 °C. The parameters employed in the DPV experiments were: potential window between 0 and 0.6 V (vs. Ag/AgCl 3M KCl), step potential 10 mV, modulation amplitude 10 mV, modulation time 0.015 s and interval time 0.1 s. 13

Figure S11. Differential pulse voltammograms (DPV) recorded for each of the four probes hybridised to the Fc-labelled ssDNA generated from blood (Sample 1) after the sensor had been exposed to each temperature starting from 25 until 40°C. The parameters employed in the DPV experiments were: potential window between 0.1 and 0.3 V (vs. Au), step potential 10 mV, modulation amplitude 10 mV, modulation time 0.015 s and interval time 0.1 s. 14

Figure S12. Comparison of the reproducibility of 9- electrode array vs 64 electrode array configurations by recording the DPVs for identically decorated electrodes with the thiolated PC and hybridised the fully complementary Fc-ssDNA (SNP G). 15

Figure S13. Study of inter-array reproducibility by comparison of the éMCA profiles obtained by plotting the maxima of the DPV recorded with T ramping (1°C /step) for blood (Sample 1) using each of the device. Three electrodes were used for constructed the melting profiles in each case. 15

Figure S14. Agarose gel (2.6 % w/v) after electrophoresis of asymmetric-RPA products obtained using real samples from fingerprick (Lines 1 to 10). Double stranded DNA was also produced using RPA in blood in a different reaction and included in the gel for comparison (Line 11). 16

Figure S15. a) Analytical data of the éMCA profiles obtained by recording DPVs with T ramping (1°C /step) and the corresponding first derivatives. Triplicates were carried out for each probe and sample used. 18

Figure S16. éMCA profiles (as % DPV signal) obtained using the second generation device by recording DPV with T ramping (1°C /step) of 10 representative blood samples and the corresponding first derivatives. Triplicates were carried out for each probe and sample used....21

References:....21

Annex:....22

Experimental Section

Reagents. All chemicals and reagents were of analytical grade and used without further purification. All solutions were prepared in ultra-pure water (18 MΩ cm) obtained using a Simplicity Water Purification System. Synthetic oligonucleotides were purchased from Biomers (Ulm, Germany). Certified molecular biology agarose gel powder, Dream Taq DNA polymerase, Lambda exonuclease, deoxynucleotide triphosphates (dNTPs) and phosphate-buffered saline (PBS) were purchased from Fisher Scientific (Madrid, Spain). GelRed™ Nucleic Acid Gel Stain (Biotium) was obtained from Biogen Científica (Barcelona, Spain). Three-millimetre thick polymethylmethacrylate (PMMA) was purchased from Industria de la Goma (Tarragona, Spain) and double-sided medical grade adhesive foil ARSeal 90880 from Adhesive Research (Ireland). RPA kits (TwistAmp® Liquid Basic kit TALQBAS01) were purchased from TwistDx, Cambridge, UK. Potassium dihydrogen phosphate (KH_2PO_4), sodium chloride (NaCl), isopropanol and potassium chloride (KCl) were purchased from Fluka. The 10-(3,5-bis((6mercaptohexyl)oxy)phenyl)-3,6,9-trioxadecanol (DT1) was purchased from SensoPath Technologies (Bozeman, MT). Oligo Clean & Concentrator Kit and DNA clean & Concentrator kit were purchased from Ecogen (Spain).

Electrode array fabrication. Nine-gold working electrode configuration: Briefly, a positive photoresist AZ1505 (MicroChemicals GmbH, Germany) was deposited by spin coating at 4000 rpm for 30 s on a pre-cleaned and dried glass slide. The photoresist was then exposed to UV light for 4 s using a chromium mask in contact mode (LED Paffrath GmbH, Rose FotoMasken, Germany) and the transferred pattern was developed using the commercial developer AZ726. Following development, the glass slide was introduced into the sputtering chamber (ATC Orion 8-HV, AJA International Inc., USA) and was subjected to an oxygen plasma etching using AC O_2/Ar (5 cm^3 s^{-1} of Ar, 5 cm^3 s^{-1} of O_2, 50 W) for 5 minutes. A layer of 30 nm of Ti/TiO_2 was then sputtered (oxygen flow rate: 5 cm^3 s^{-1} of O2 for the first 10 nm, then increased to 20 cm^3 s^{-1} for the last 5 nm. Ar flow rate: constant 5 $cm^3 s{-}1$). This was followed by the deposition of 100 nm of Au by AC sputtering (5 cm^3 s^{-1} of Ar, 5 cm^3 s^{-1} of O_2, 50 W). Lift-off was achieved using sonication in acetone for 5 minutes, then 5 minutes in isopropanol and finally rinsing with Milli-Q water.

Electrochemical cleaning of electrode array and probe immobilisation. The array of 9 working gold electrodes and a common gold counter electrode were used with a classical reference electrode Ag/AgCl. Working electrodes were cleaned by sequential sonication in isopropanol, acetone and water for 5 minutes each, followed by electrochemical cleaning by cycling 10 times, firstly in 0.5 M KOH between 0.0 and -1.2 V vs Ag/AgCl, and secondly in 0.5 M sulfuric acid between 0.0 and 1.6 V vs Ag/AgCl at a scan rate of 100 mVs^{-1}. Washing steps in Milli-Q water were included in between both cleaning procedures and again at the end. After drying with nitrogen, the electrodes were immediately used for surface functionalization.

In the case of the screen-printed 64 electrode array, the cleaning procedure was significantly simpler based on sequential washing steps with 50 % v/v isopropanol in water, and water. After drying with nitrogen, the electrodes were immediately used for surface functionalisation.

Thiolated capture probes (Table S1) (probe-A, probe-T, probe-C and probe-G) were self-assembled on each of the individual electrodes in duplicate, by spotting 1 µL of 1 µM thiolated capture probe solution + DT1 as backfiller with a 1:100 Probe:DT1 molar ratio freshly prepared in 1 M KH_2PO_4. This functionalization solution was left to assemble at 37°C for 3 h in humidity chamber to avoid evaporation, followed by a thorough wash with Milli-Q water and finally dried with nitrogen.

Sequences design. dbSNP database from National Center for Biotechnology Information (NCBI) was used to obtain the osteoporosis sequence related with SNP rs2741856. For the melting curve analysis a 138-mer synthetic target containing the SNP site and four probes sequences in such a way to capture the SNP in the target at the middle according to previous work[1] were designed (Table S1). Moreover, to mimic the genomic DNA, dsDNA should be produced and, specific primers to the synthetic 138-mer sequence were designed using Primer Blast software (Table S2). For electrochemical detection the redox label ferrocene (Fc) was introduced in the amplified product using a Fc-labelled forward primer in an RPA reaction.

Preparation of the dsDNA from synthetic 138-mer ssDNA by PCR, for further RPA amplification. A dsDNA was produced to mimic genomic DNA. This dsDNA was produced by PCR from a synthetic 138-mer sequence (Table S2). The PCR was carried out using a T100 thermal cycler (Biorad) following the protocol: 95°C for 2 minutes, followed by 25 cycles at 95°C for 30 seconds, 60°C for 30 seconds, and 72°C for 30 seconds, with a final elongation step at 72°C for 5 minutes. One hundred microliters of PCR reaction mixture contained 1 unit of DreamTaq and DreamTaq buffer 1X, both forward and reverse primers without modifications at 200 nM, dNTPs at 200 μM and 100 pM synthetic 138-mer DNA as final concentrations. A DNA Clean & Concentrator™ kit was used to purify the products from the reaction mixture and the amplification products were visualized using agarose gel electrophoresis as described below. The concentration of the amplicon was measured using a SimpliNano spectrophotometer (Fisher Scientific, Spain).

Gel electrophoresis Analysis. For gel electrophoresis analysis, a gel was made with certified molecular biology agarose gel powder (2.6% w/v) in 1× Tris-Borate- EDTA buffer (TBE) and stained with GelRed™ nucleic acid stain. A mixture of 5 μL of asymmetric-RPA product with 4 μL of loading buffer 6x was loaded per gel well and the electrophoresis was performed at 100 V, for 30 min and gels were visualized in a UV transilluminator. Finally, the samples were used immediately or stored at -20 °C until use. For better understanding of the gel, Image J software was used to calculate the intensity of the band and the obtained values were normalised by percentage and ploted in a graph.

Assymmetric RPA reaction. Two optimised conditions were carried out:

1) Reaction time optimisation for asymmetric RPA for single stranded DNA generation. A common master mix containing Fc-FwP (1000 nM) and reverse primer (200 nM) were prepared and aliquoted in five vials and the RPA reaction carried out for different times (5, 10, 15, 20 and 30 min).

2) Primer ratio optimisation for Asymmetric RPA for single stranded DNA generation. Using 15 min amplification, different ferrocene labelled forward primer:reverse primer (Fc-FwP:RevP) molar ratios were evaluated to find the optimum conditions for amplification and subsequent electrochemical detection. The reverse primer concentration was kept constant at 200 nM and the Fc-FwP concentrations (200, 500, 1000, 2000 and 5000 nM) was consecutively increased, resulting in Fc-FwP:RevP molar ratios of 1:1, 2:1, 5:1, 10:1 and 25:1 (Table S3).

Tables

Table S1. Sequences of target and capture probes related with SNP used for éMCA (each one has a SNP in the middle). The regions of the target sequence complementary to the capture probes are underlined) and also the fully complementary capture probe: Probe-C. SNP-GG related to osteoporosis.

Osteoporosis Target (rs2741856) (138-mer ssDNA)	5′- CACACACACACCCCTCTTCACTATAATTATAATTACTATG TTGGCTTCCAGATCAGGGGTTAGAGCCTTGGCATGGAGAC GCCTGAAAGGCACCCAAGGCAATTAGTGGTGTCCCTTC TCCACCCCCTACATACCTTC - 3′
Two specific probes related to the SNP used for éMCA (21-mer)	
probe-C (PC)	5’- AACCCCTGAT**C**TGGAAGCCAA-thioctic acid -3
probe-G (PG)	5’- AACCCCTGAT**G**TGGAAGCCAA-thioctic acid -3
Two probes used as negative controls related to the SNP used for éMCA (each one has a non-related SNP base at the middle).	
probe-A (PA)	5’- AACCCCTGAT**A**TGGAAGCCAA-thioctic acid -3
probe-T (PT)	5’- AACCCCTGAT**T**TGGAAGCCAA-thioctic acid -3

Table S2. Primers for dsDNA generation by PCR and for RPA reaction

Osteoporosis Target (rs2741856) 138-mer ssDNA	5′- CACACACACACCCCTCTTCACTATAATTATAATTACTATG TTGGCTTCCAGATCAGGGGTTAGAGCCTTGGCATGGAGAC GCCTGAAAGGCACCCAAGGCAATTAGTGGTGTCCCTTC TCCACCCCCTACATACCTTC - 3′
Forward primer used to produce dsDNA for further asymmetric RPA amplification (to simulate a real sample)	5´CACACACACACCCCTCTTCACTATAAT- 3′
Reverse primer (RevP)	5′- GAAGGTATGTAGGGGGTGGAGAAG- 3′
Ferrocene-Forward primer used to generate the Ferrocene-labelled ssDNA in the asymmetric RPA amplification (Fc-FwP)	5′-Fc-CACACACACACCCCTCTTCACTATAAT- 3′
Phosphorylated reverse primer used for Lambda exonuclease treatment.	5′-pho-GAAGGTATGTAGGGGGTGGAGAAG- 3′

Table S3. Ratio between forward and reverse primers used for optimisation of the asymmetric-RPA

**Primers used**	**Final concentration of primers (nM)**				
	**1**	**2**	**3**	**4**	**5**
Fc-FwP	200	500	1000	2000	5000
RevP	200	200	200	200	200
FwP/RevP ratio	1	2.5	5	10	25

Table S4. Correlation between the SNP detected by the éMCA method and results obtained using Sanger sequencing[40] (used as a reference method).

**Sample**	**éMCA**	**Sanger Sequencing**
1	GG	GG
2	CC	CC
3	GC	GC
4	GG	GG
5	GG	GG
3	GC	GC
7	GG	GG
8	GG	GG
9	GG	GG
10	GG	GG

Figures

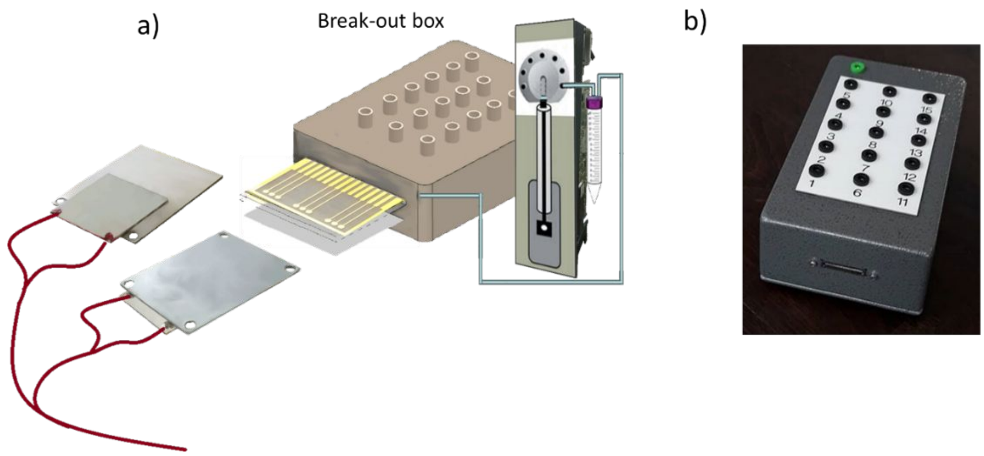

Figure S1. (a) First generation of the set-up; (b) Break-out box developed by Labman for this project.

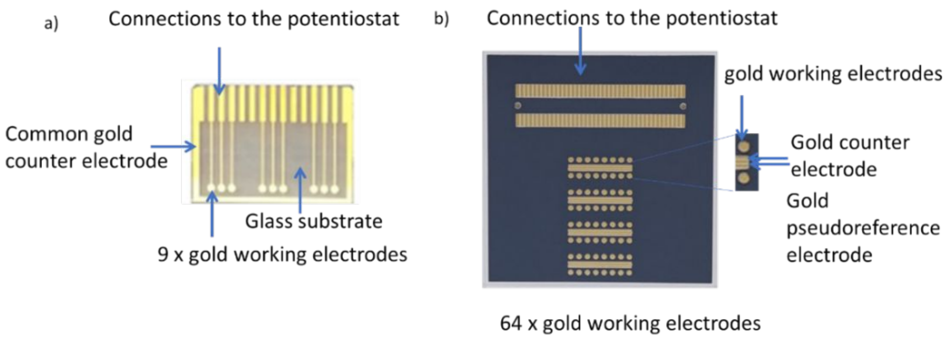

Figure S2. Electrode arrays used in the present work (a) 9 x gold working electrode array and (b) 64 x gold working array.

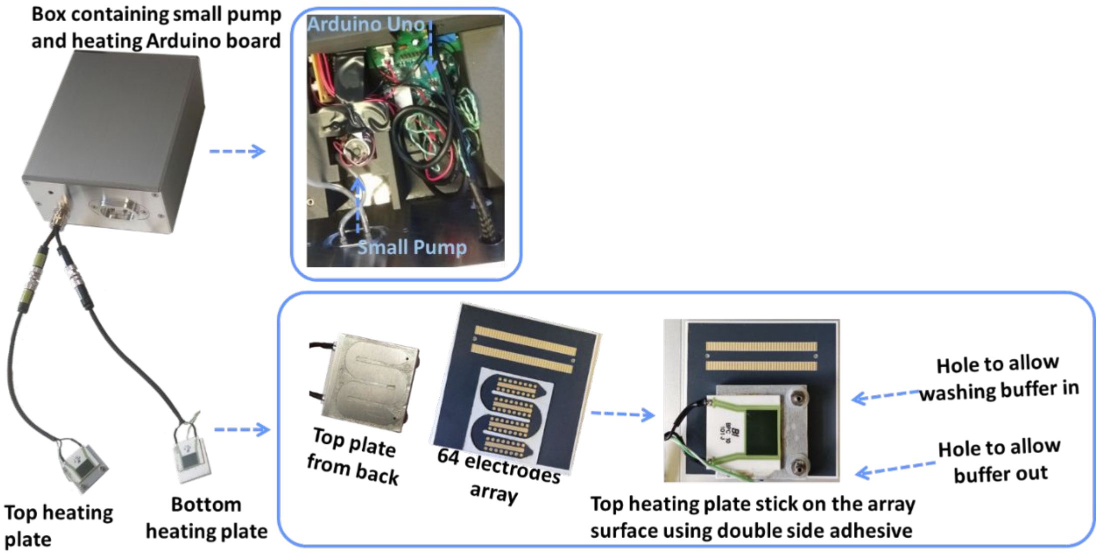

Figure S3. Real picture of the box containing a small pump and Arduino board with the internal view in the inset. The assembly of the 64 x working electrode array for the melting procedure for the second generation device.

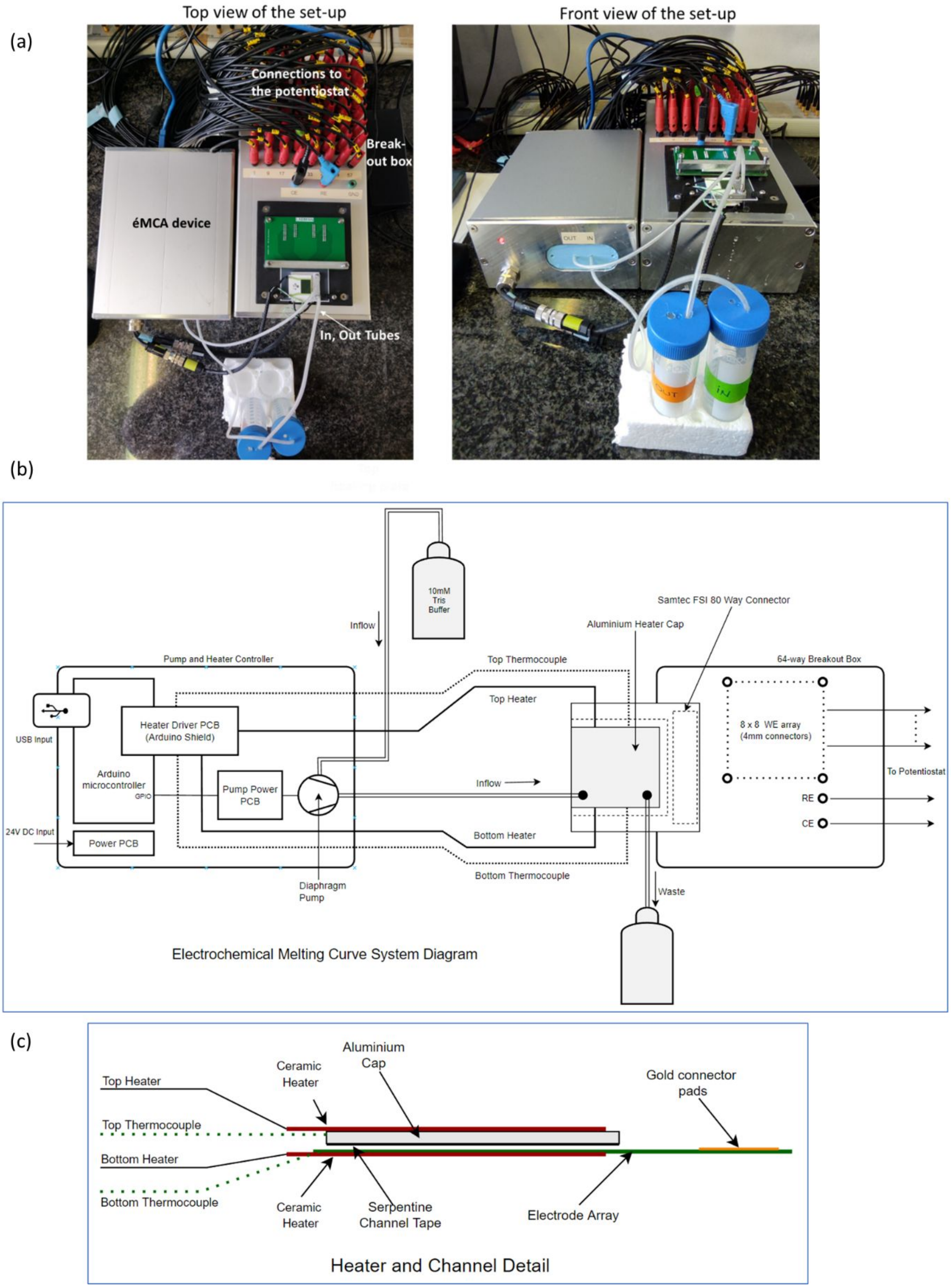

Figure S4. (a) Top and front views of the second generation of the set-up developed for the present work.(b) Electrochemical Melting Curve System diagram. (c) Heater and channel detail.

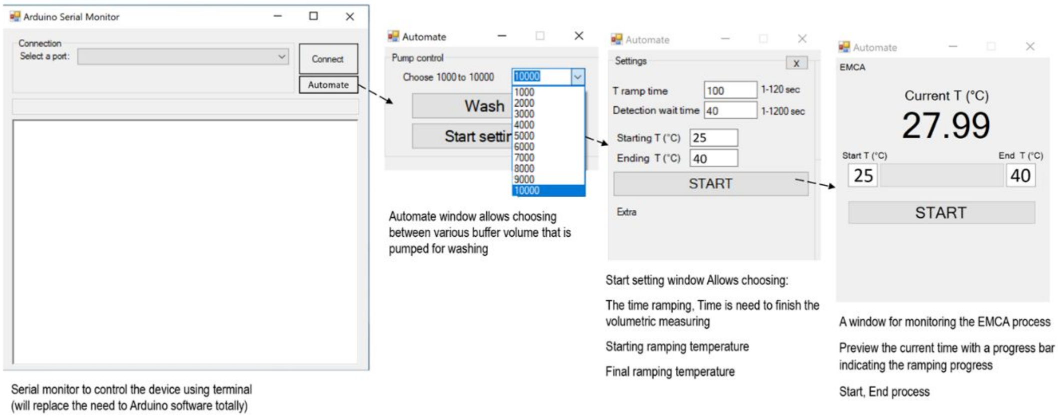

Figure S5. Screenshot of the software specifically developed to run the electrochemical melting curve analysis prototype showing how the flow rate of the washing buffer and the rate of the temperature ramps are controlled, and the temperature continuously measured throughout.

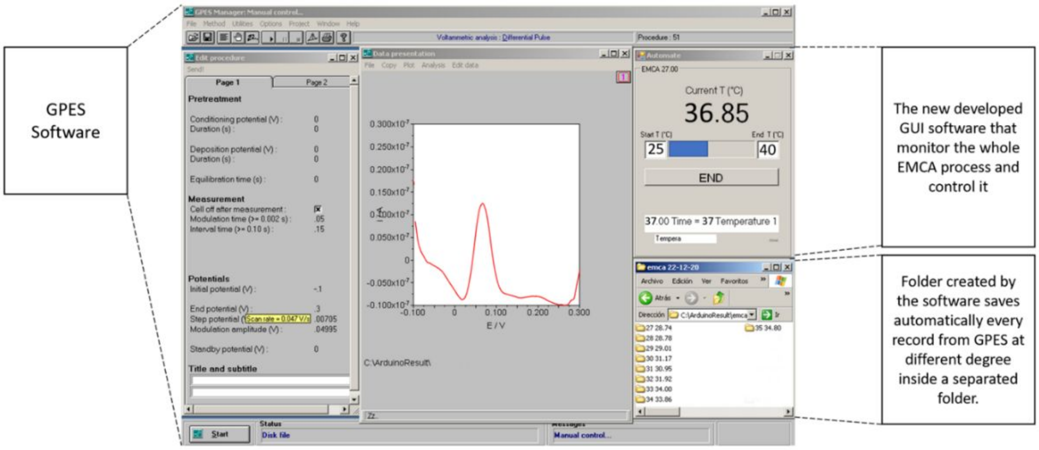

Figure S6. Screenshot of the continuous DPV measurement throughout the temperature ramp.

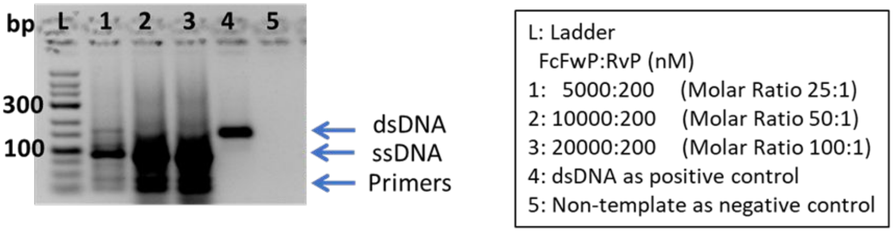

Figure S7. Preparation of ssDNA with a higher excess of Fc-Forward primer than the used in Figure 3 of the manuscript (2.6 % w/v agarose gel after electrophoresis of asymmetric-RPA products obtained using PCR generated dsDNA as target)

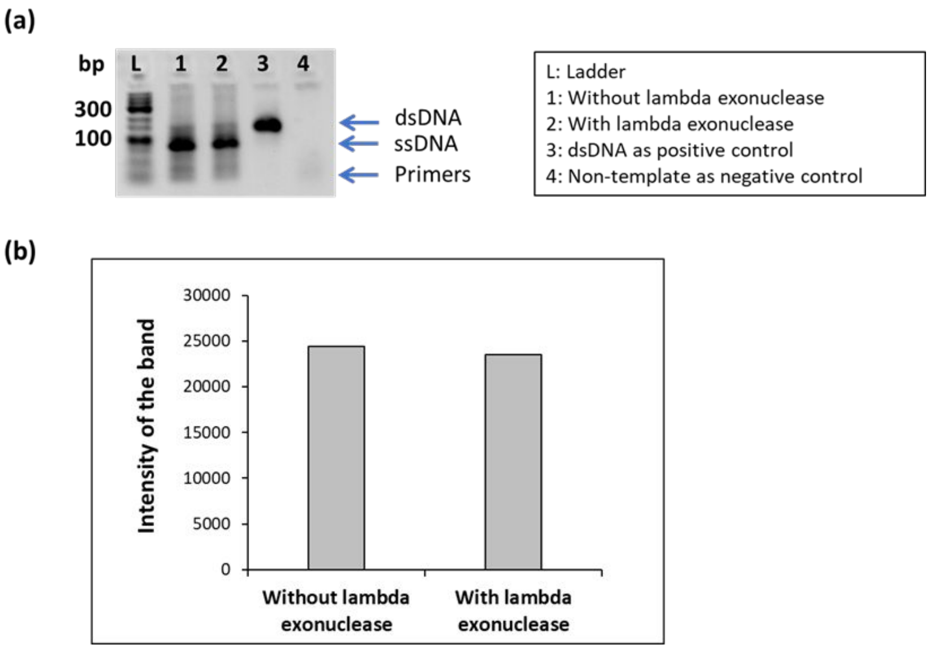

Figure S8. Preparation of ssDNA with and without lambda exonuclease digestion: (a) 2.6 % w/v agarose gel after electrophoresis of asymmetric-RPA products obtained using PCR generated dsDNA as target and with and without the addition of lambda exonuclease; (b) the graph obtained using Image J software to calculate the intensity of the band.

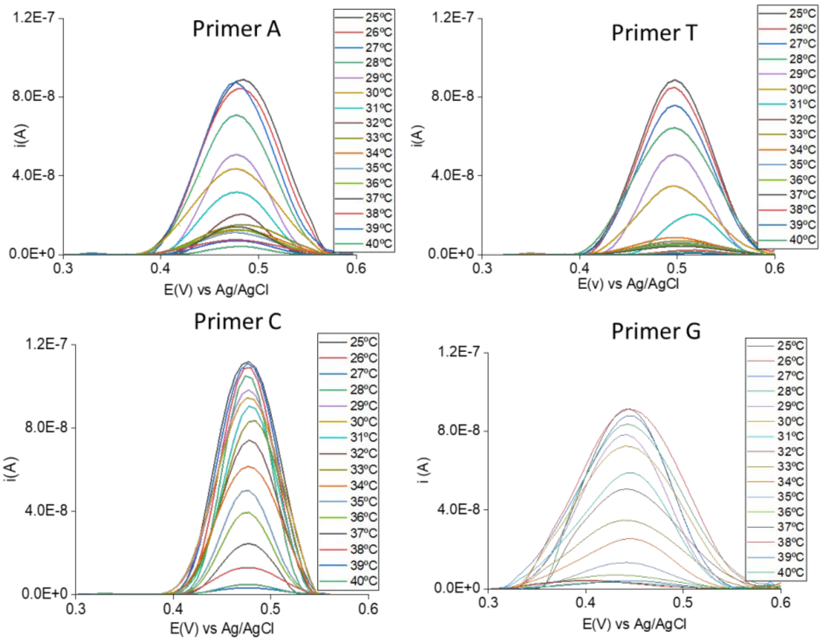

Figure S9. Differential pulse voltammograms (DPV) recorded for each of the four probes hybridised to the Fc-labelled synthetic target after the sensor had been exposed to each temperature starting from 25 until 40 °C. The parameters employed in the DPV experiments were: potential window between 0 and 0.6 V (vs. Ag/AgCl 3M KCl), step potential 10 mV, modulation amplitude 10 mV, modulation time 0.015 s and interval time 0.1 s.

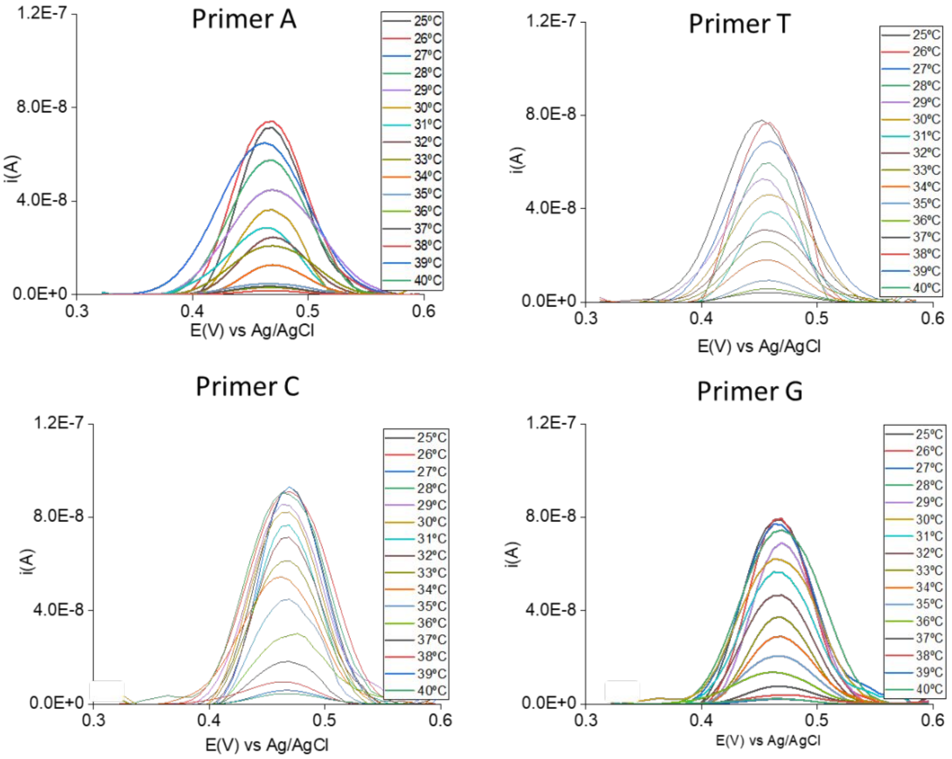

Figure S10. Differential pulse voltammograms (DPV) recorded for each of the four probes hybridised to the Fc-labelled ssDNA generated from blood (Sample 1) after the sensor had been exposed to each temperature starting from 25 until 40 °C. The parameters employed in the DPV experiments were: potential window between 0 and 0.6 V (vs. Ag/AgCl 3M KCl), step potential 10 mV, modulation amplitude 10 mV, modulation time 0.015 s and interval time 0.1 s.

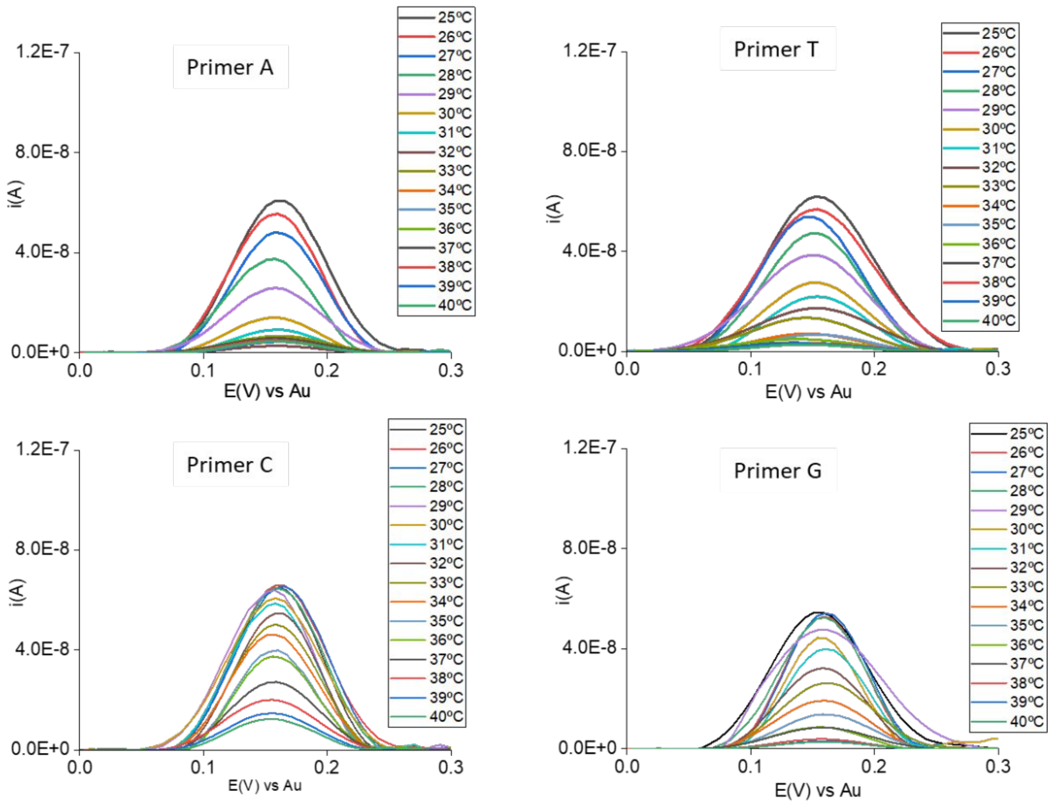

Figure S11. Differential pulse voltammograms (DPV) recorded for each of the four probes hybridised to the Fc-labelled ssDNA generated from blood (Sample 1) after the sensor had been exposed to each temperature starting from 25 until 40°C. The parameters employed in the DPV experiments were: potential window between 0.1 and 0.3 V (vs. Au), step potential 10 mV, modulation amplitude 10 mV, modulation time 0.015 s and interval time 0.1 s.

Each of the DPV curves in Figures S9 to S11 represents the DPV taken at different temperatures, as indicated in the inset on the right handside, with decreasing peaks, and peak currents due to the thermally induced dissociation of the ferrocene labelled target ssDNA. The raw data taken using the DPV measurement at the different temperatures are then used to create a malting curve for the determination of the melting temperature. Firstly, the baseline correction of DPV peaks was done using the GPES program. After that, the average of the maximum height (maximum current) of each replicate of DPV curves at each temperature was calculated. Finally, taking the DPV carried out at the starting temperature as 100%, the percentage of the DPV signal at each temperature was calculated and the melting profiles were constructed by plotting these percentages vs temperature. For fitting the curves, the mathematic Biphasic Dose Response Function was applied using the Levenberg Marquardt iteration algorithm and the melting temperatures were obtained from the extrema (maximum) of their first derivative.

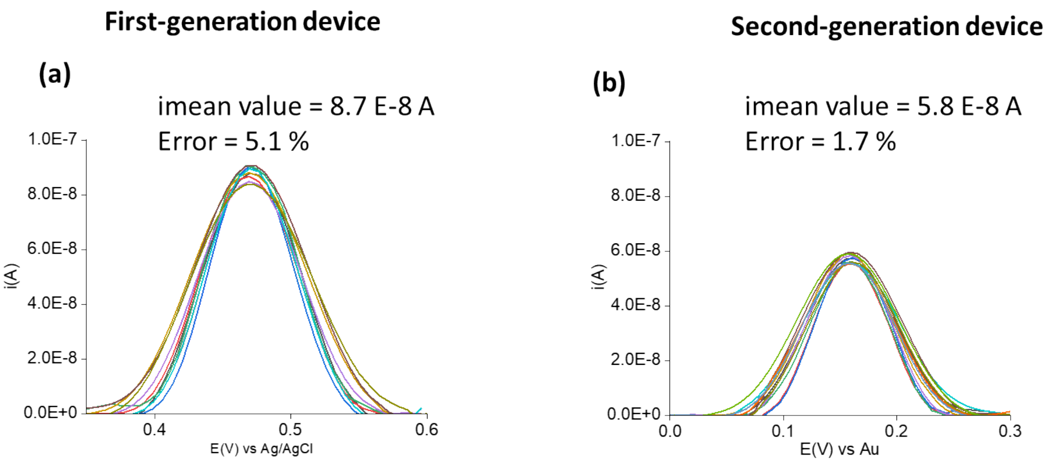

Figure S12. Comparison of the reproducibility of the (a) 9- electrode array vs the (b) 64 electrode array configurations by recording the DPVs for identically decorated electrodes with the thiolated PC and hybridised the fully complementary Fc-ssDNA (SNP G).

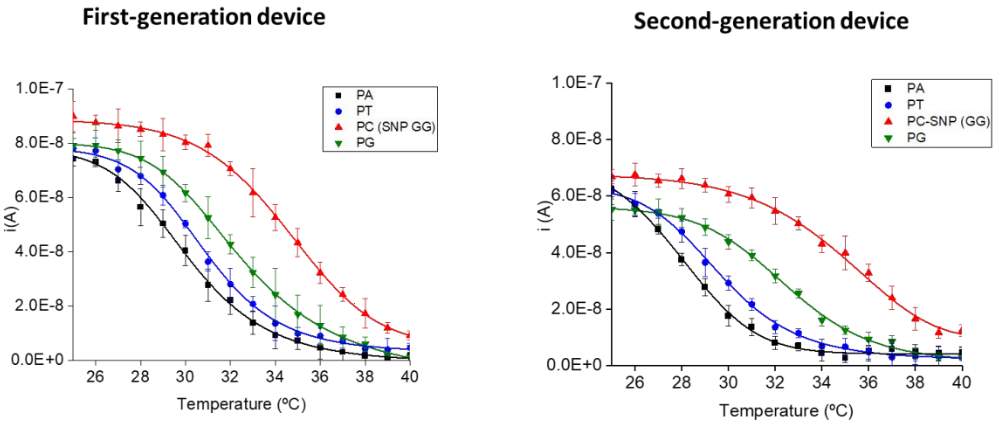

Figure S13. Study of inter-array reproducibility by comparison of the éMCA profiles obtained by plotting the maxima of the DPV recorded with T ramping (1°C /step) for a fingerprick blood sample (Sample 1) using each of the devices. Three electrodes were used for constructing the melting profiles in each case.

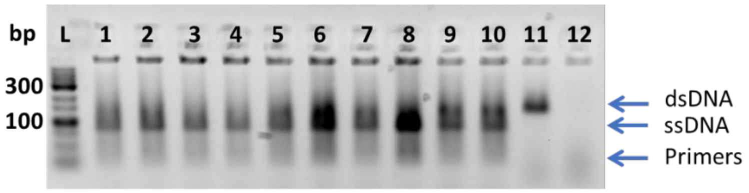

L: Ladder
1: Sample 1
2: Sample 2
3: Sample 3
4: Sample 4
5: Sample 5
6: Sample 6
7: Sample 7
8: Sample 8
9: Sample 9
10: Sample 10
11: dsDNA as positive control
12: Non-template control as negative control

Figure S14. Agarose gel (2.6 % w/v) after electrophoresis of asymmetric-RPA products obtained using real blood samples, with Sample 1 being a fingerprick blood sample and Samples 2-10 being biobank venous blood samples (Lines 1 to 10). Double stranded DNA was also produced using RPA in blood in a different reaction and included in the gel for comparison (Line 11).

Raw data 1st generation device

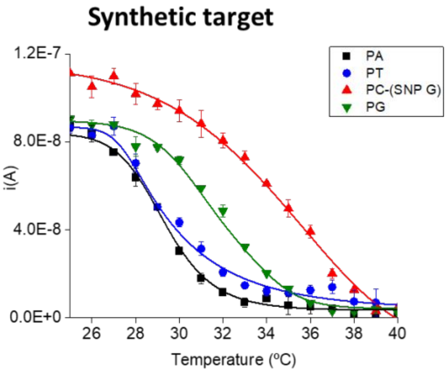

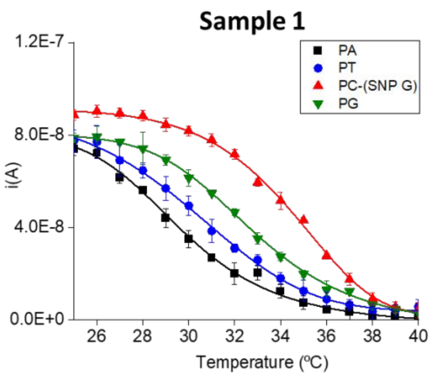

Raw data 2nd generation device

Sample 1

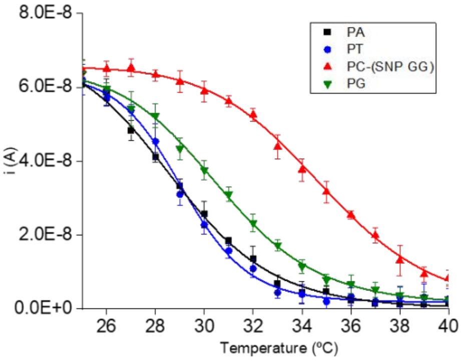

Sample 2

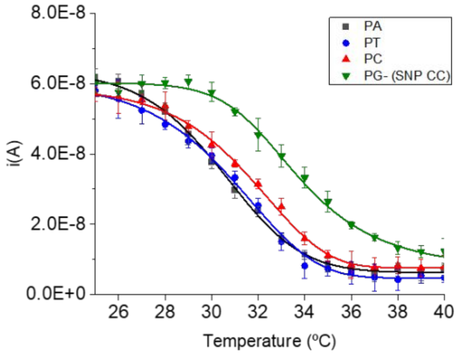

Sample 3

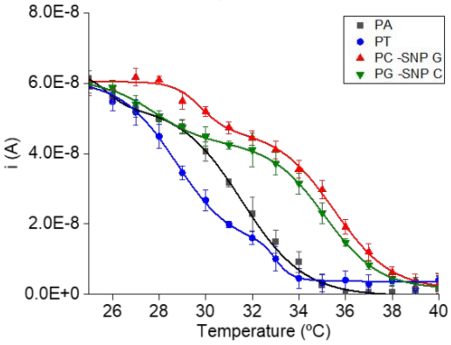

Sample 4

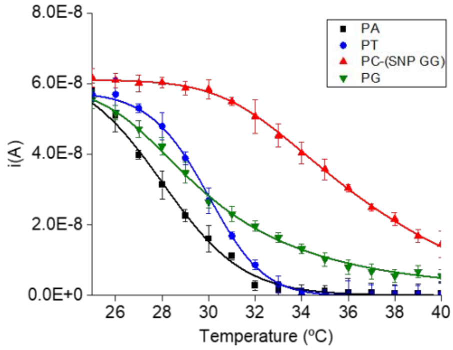

Sample 5

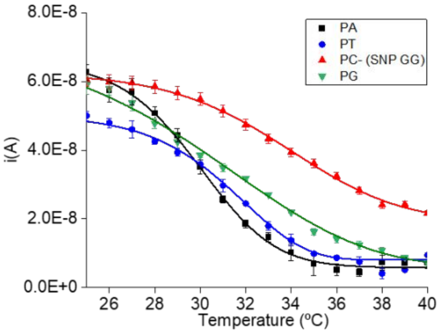

Sample 6

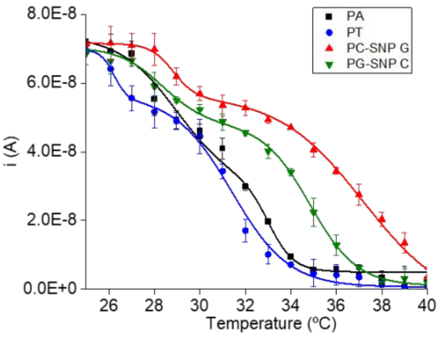

Sample 7

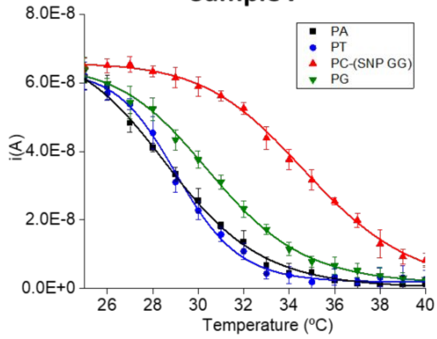

Sample 8

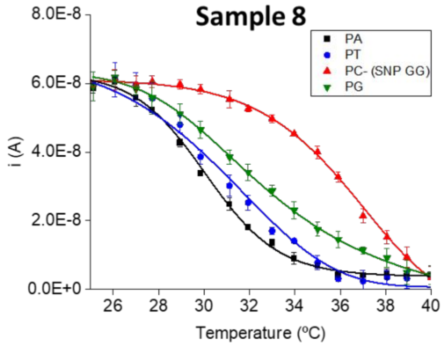

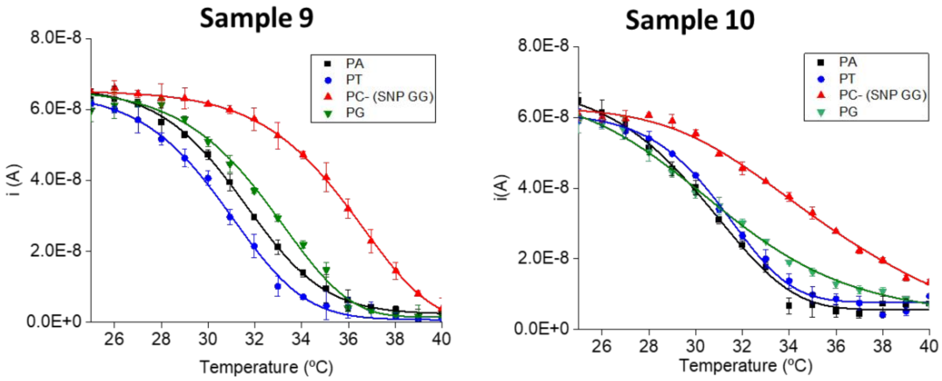

Model: Biphasic Dose Response Function

Equation double span = A2 - A1;

double Section 1 =span*p/(1+pow(10,(LOGx01-x)*h1));

double Section 2 =span* (1-p)/(1+pow(10,(LOGx02-x)*h2));

y=A1 + Section1 +Section2

Iteration algoristhm: Levenberg Marquardt

Square correlation coeficient = R^2

**Sample**	**R^2 PA**	**R^2 PT**	**R^2 PC**	**R^2 PG**
Synthetic DNA (1st device)	0.9971	0.9943	0.9946	0.9991
Sample 1 (1st device)	0.9986	0.9984	0.9988	0.9996
Sample 1 (2nd device)	0.9981	0.9974	0.9988	0.9987
Sample 2	0.9984	0.9975	0.9981	0.9971
Sample 3	0.9986	0.9988	0.9967	0.9996
Sample 4	0.9994	0.9992	0.9995	0.9997
Sample 5	0.9952	0.9972	0.9943	0.9951
Sample 6	0.9989	0.9984	0.9975	0.9995
Sample 7	0.9981	0.9974	0.9988	0.9987
Sample 8	0.9945	0.9979	0.9976	0.9974
Sample 9	0.9989	0.9997	0.9988	0.9995
Sample 10	0.9988	0.9984	0.9967	0.9985

Figure S15. a) Analytical data of the éMCA profiles obtained by recording DPVs with T ramping (1°C /step) and the corresponding first derivatives. Triplicates were carried out for each probe and sample used.

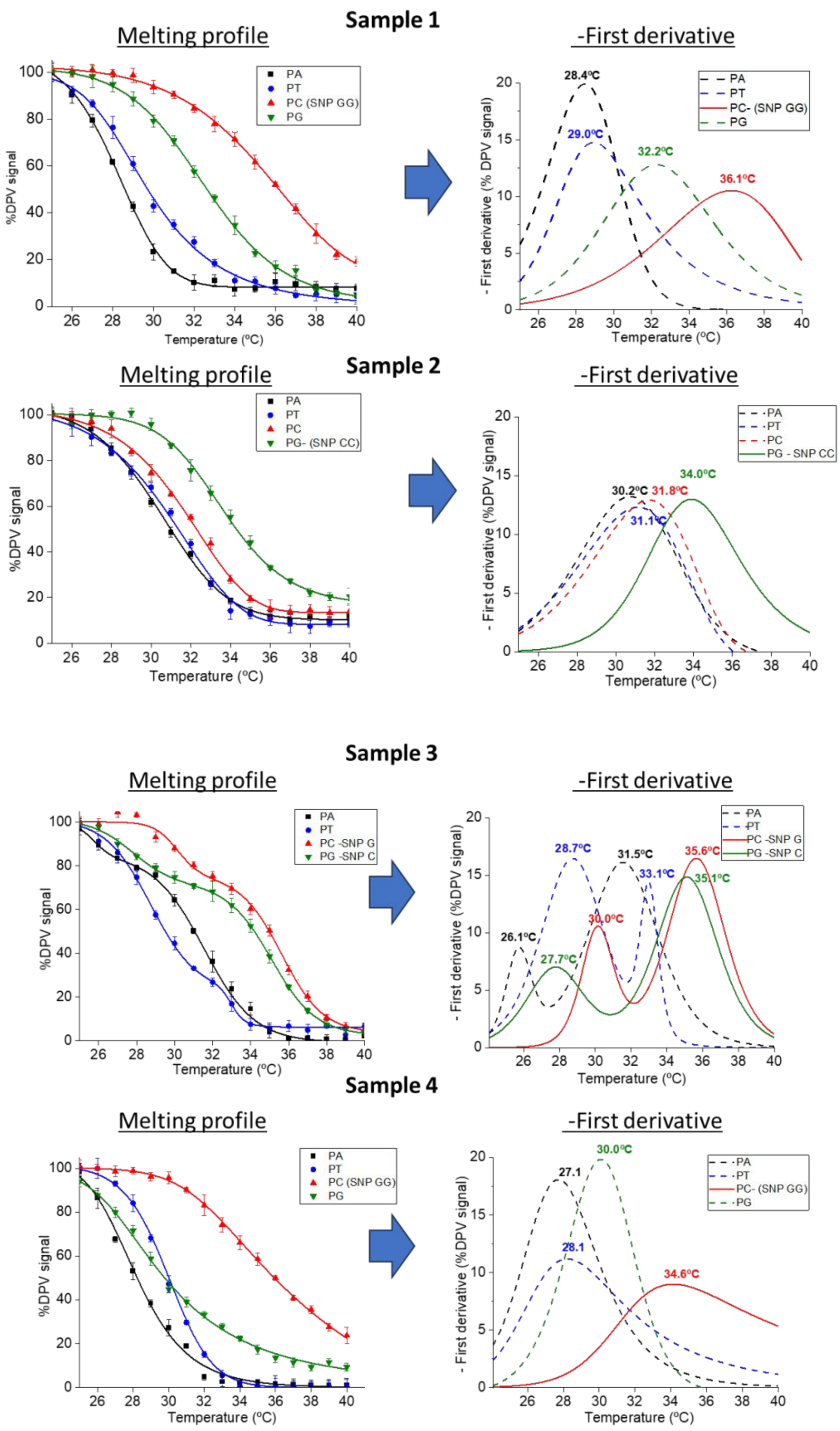
Sample 1
Melting profile
-First derivative
Sample 2
Melting profile
-First derivative
Sample 3
Melting profile
-First derivative
Sample 4
Melting profile
-First derivative
%DPV signal
Temperature (ºC)
- First derivative (% DPV signal)
PA
PT
PC (SNP GG)
PG
PC- (SNP GG)
PG- (SNP CC)
PG - SNP CC
PC -SNP G
PG -SNP C
28.4ºC
29.0ºC
32.2ºC
36.1ºC
30.2ºC
31.8ºC
31.1ºC
34.0ºC
26.1ºC
28.7ºC
31.5ºC
33.1ºC
35.6ºC
35.1ºC
30.0ºC
27.7ºC
27.1
28.1
30.0ºC
34.6ºC

Sample 5

Melting profile — -First derivative

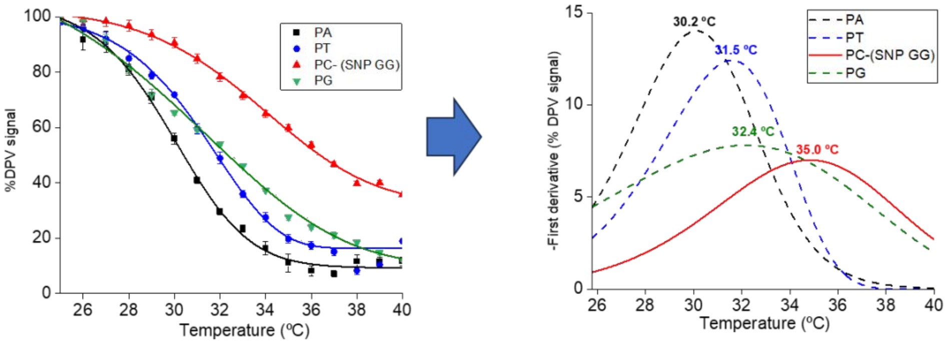

Sample 6

Melting profile — -First derivative

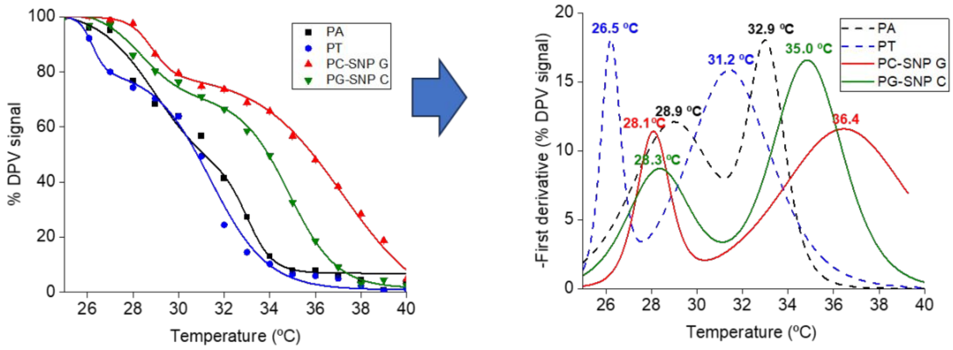

Sample 7

Melting profile — -First derivative

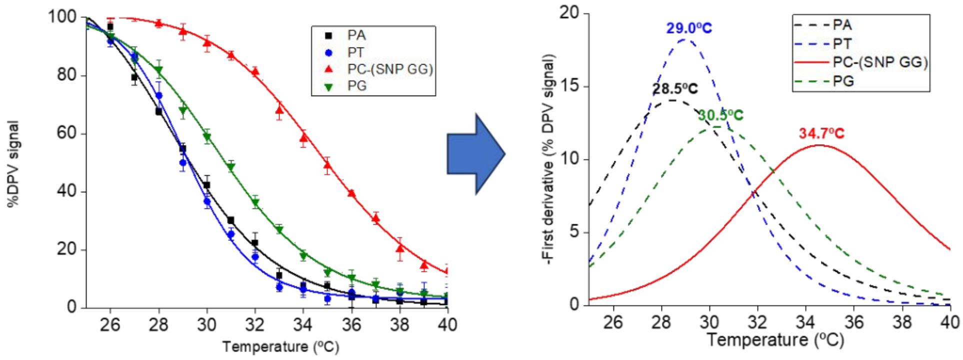

Sample 8

Melting profile — -First derivative

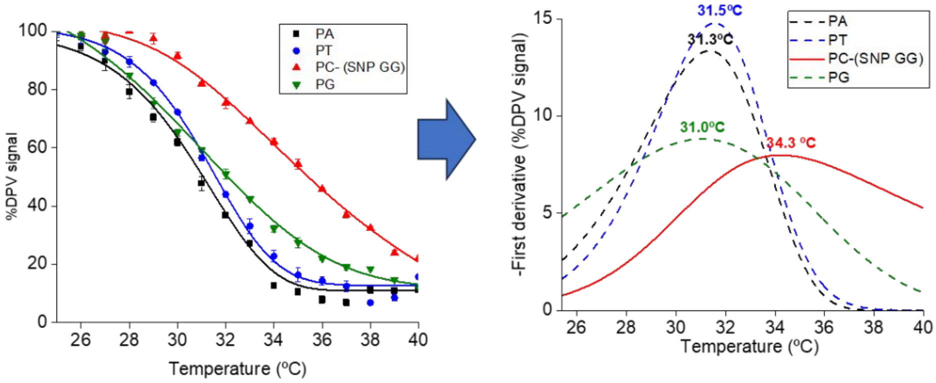

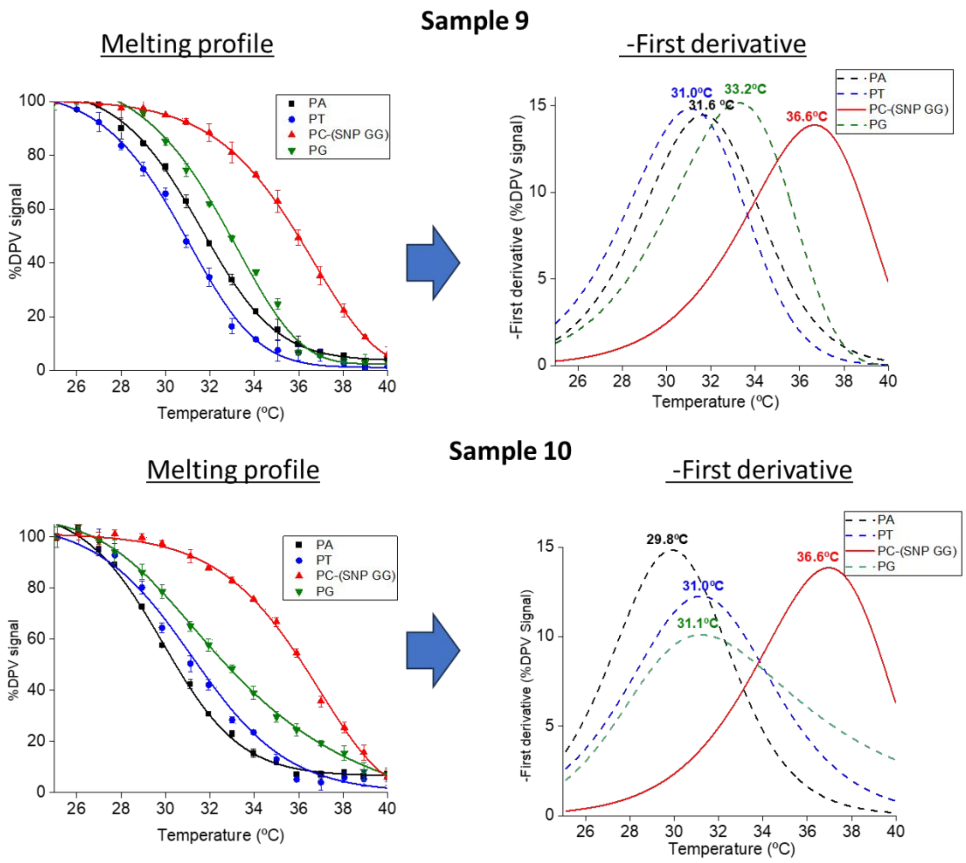

Figure S16. éMCA profiles (as % DPV signal) obtained using the second-generation device by recording DPV with T ramping (1°C /step) of 10 representative blood samples and the corresponding first derivatives. Triplicates were carried out for each probe and sample used.

References:

(1) Chahin N.; Escobar-Nassar S.; Osma J.; Bashammakh A. S.; AlYoubi A. O.; Ortiz M.; O'Sullivan C. K. Low-Cost Platform for Multiplexed Electrochemical Melting Curve Analysis. ACS Meas. Sci. Au, 2022, 2, 147–156.

Annex:

User Manual for the second generation device

This document provides notes for use of the pump and heater box designed primarily for use with 80 pad electrode arrays. The device is intended to be used with a 64-way breakout box as shown below though can be used with other sensor test rigs. The device houses an Arduino, a heater shield, a small diaphragm pump, and supporting electronics. The Arduino can be connected to via the USB port at the back of the machine via a serial connection at 115200 baud with no CR or NL. Through serial commands, the device can automate the SNP detection process, with tightly controlled temperature ramping from 20 to 60°C.

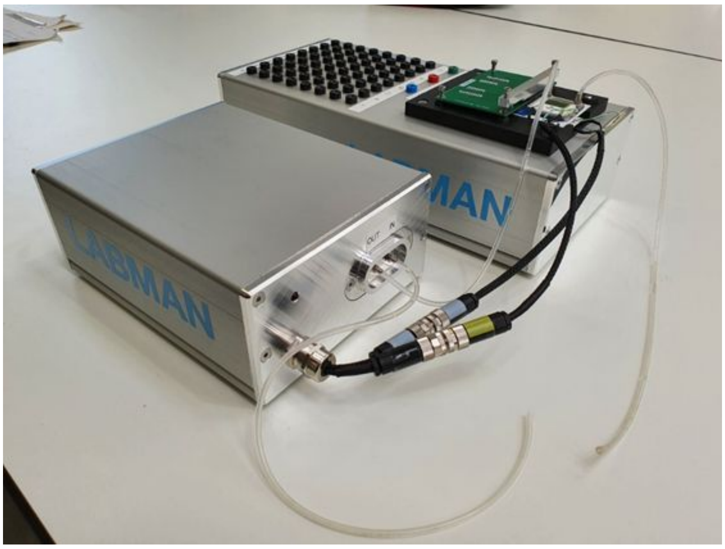

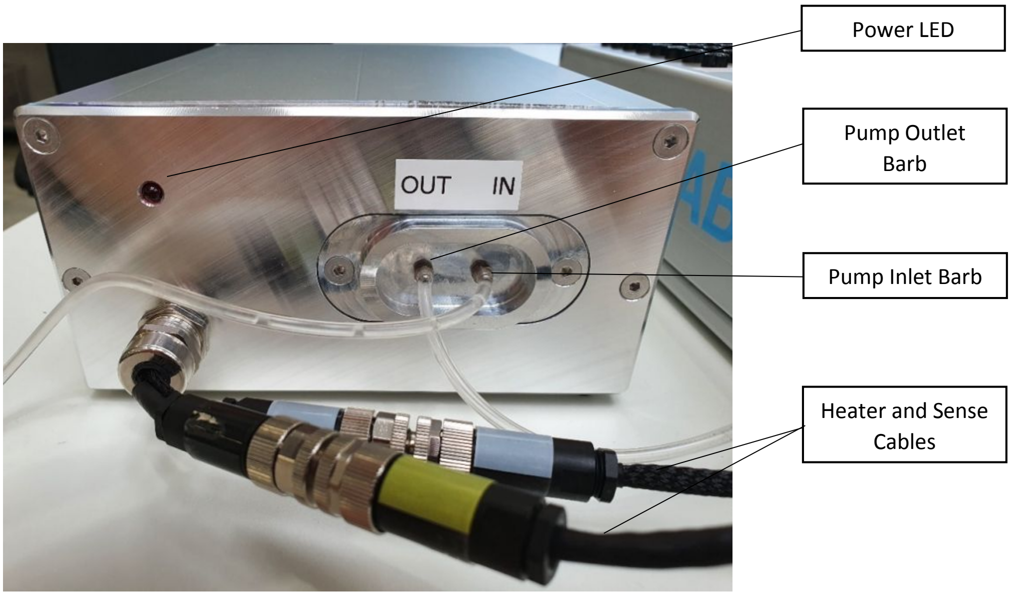

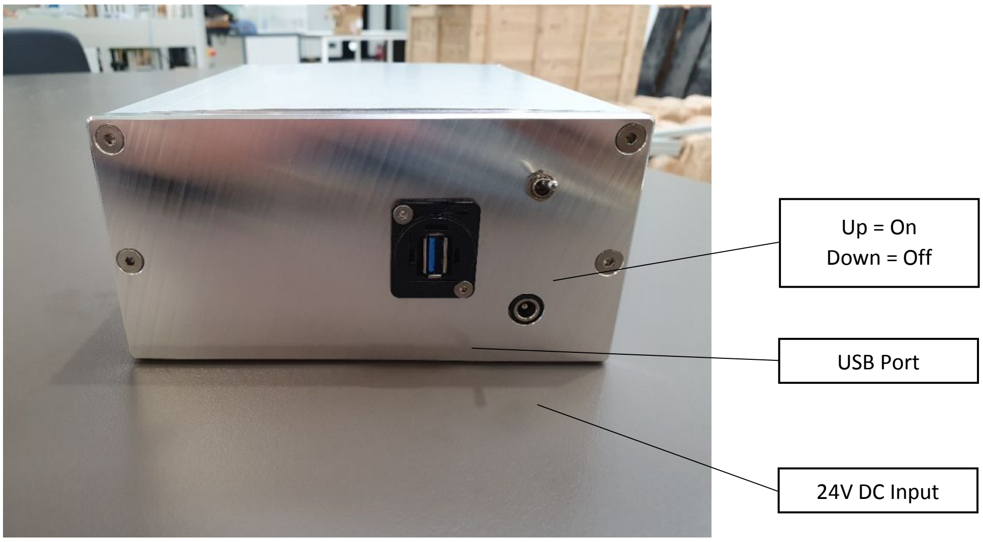

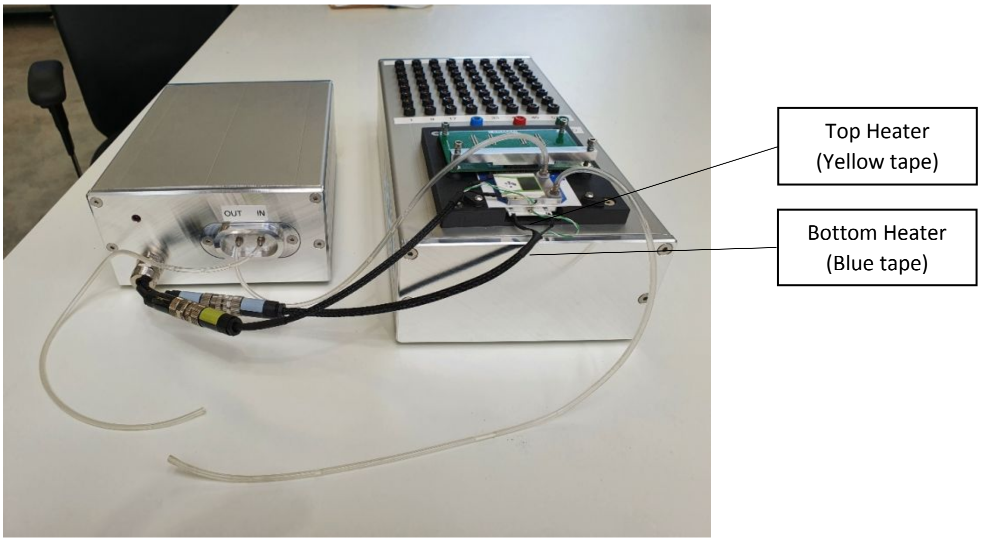

Setup:

The photo above demonstrates how the system should be setup to work with the 64-way breakout box. The input barb of the system box is ready to be plumbed into a vessel of feed liquid, and the output barb is plumbed into one of the two ports on the top heater. The remaining barb on the aluminium plate is ready to be plumbed into a waste liquid vessel.

The top heater is fixed to the ceramic electrode array with specially cut double sided tape. This tape has a serpentine channel cut out of it so that the liquid will pass from one barbed port, over the electrodes, and out of the other barbed port. The bottom plate sits under the ceramic electrode array and helps to mitigate heat loss to the environment at higher operating temperatures.

The Process:

Initial Dispense:

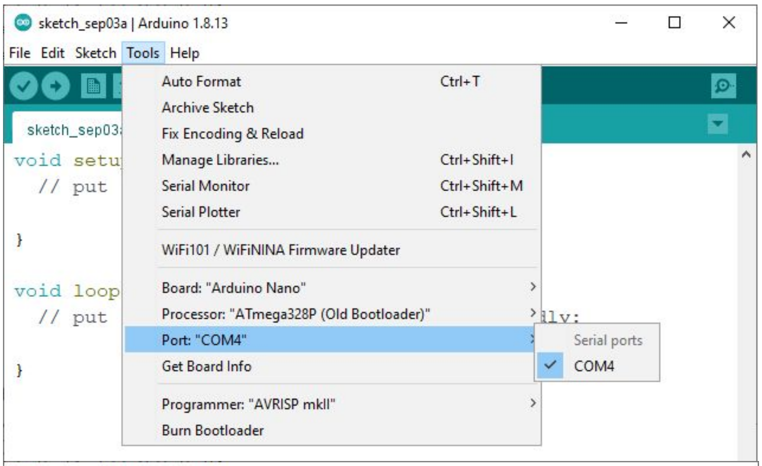

To communicate with the device, you must first establish a serial connection. Install and run the Arduino IDE, then plug the device into your computer via USB.

Navigate to "Tools" and then select whichever port the device has connected to on your PC (this differs from PC to PC).

It is recommended to use the Arduino serial terminal, but if you are confident with another terminal or wish to integrate it into your own software, feel free to do so.

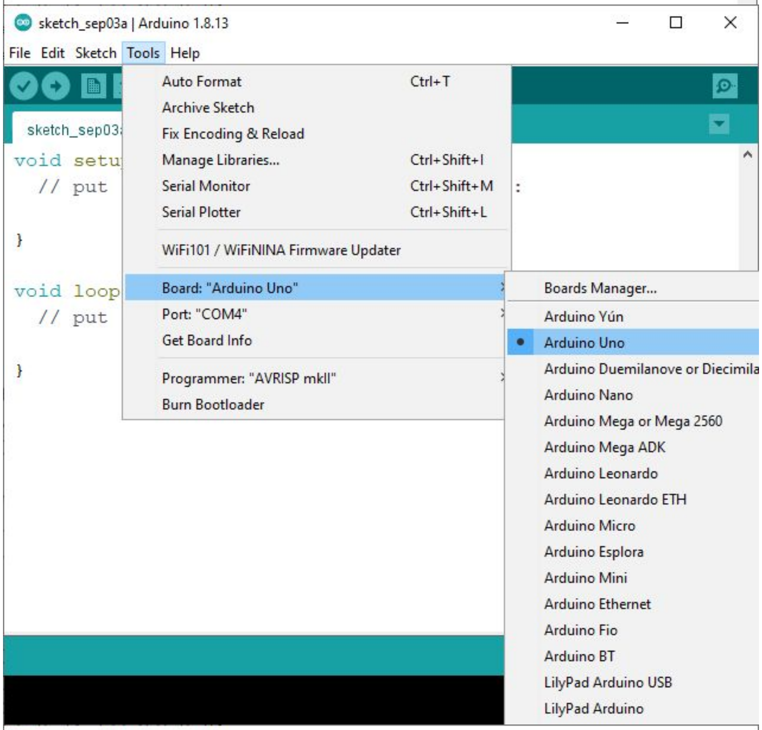

Next, select Arduino Uno as the Board.

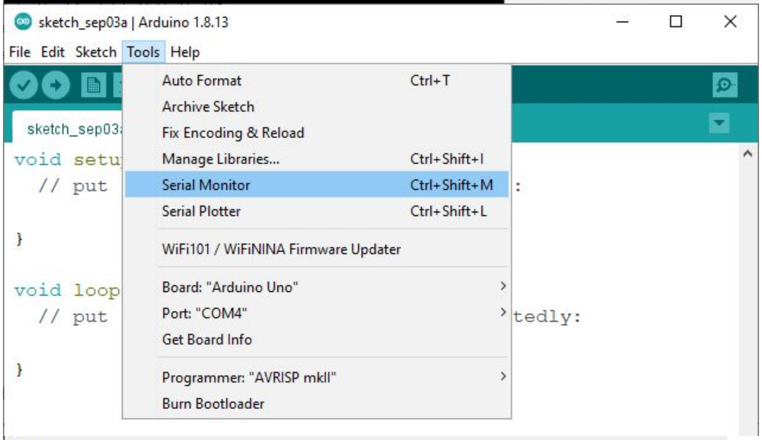

Finally, navigate to the "Tools" menu and select "Serial Monitor".

In the bottom right corner of the serial monitor, set the baud rate to 115200 baud. Set the carriage return/new line drop down box to "No line ending".

Initial Dispense:

Upon establishing a serial connection, the user will be

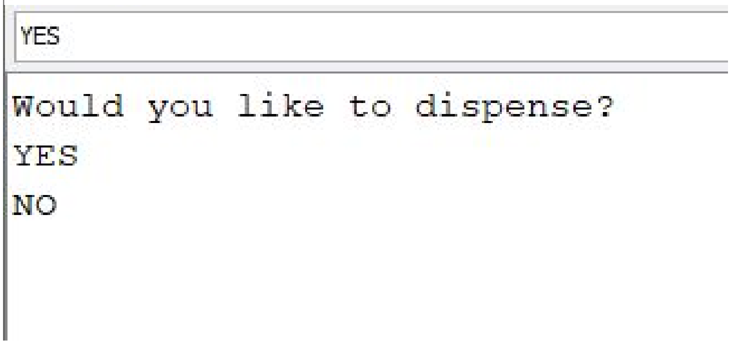

presented with the initial dispense menu.

In the first screenshot, it can be seen that “YES” has been typed into the top of the serial monitor. Upon pressing send or the enter key, this text will be sent to the device.

The second picture shows that the device has registered the “YES” command, as it has repeated it back and progressed to the next prompt.

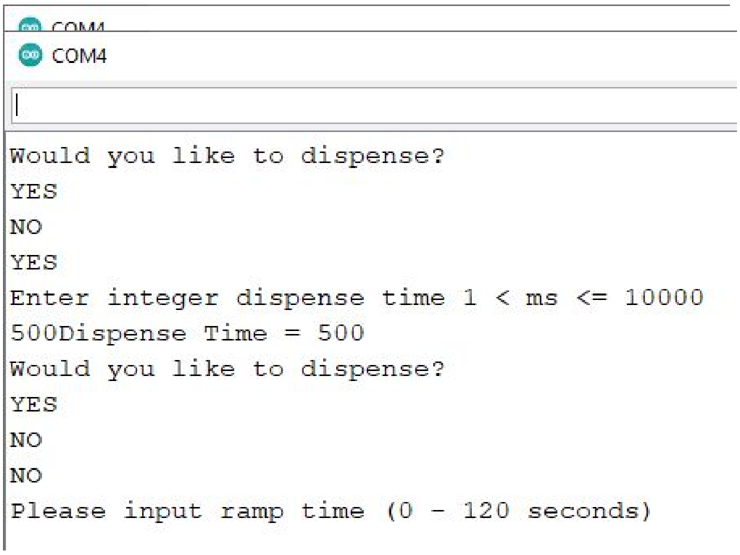

The user will be asked whether they would like to dispense liquid for 1ms – 10000ms. This allows the liquid being tested to be progressed through the piping, to the chip. By following the on-screen prompts, the user can run this initial dispense as many times as they desire before starting the main process. Once the initial dispense menu has been exited, the device must be restarted to regain access to this feature.

Ramp and Detection Time Setup:

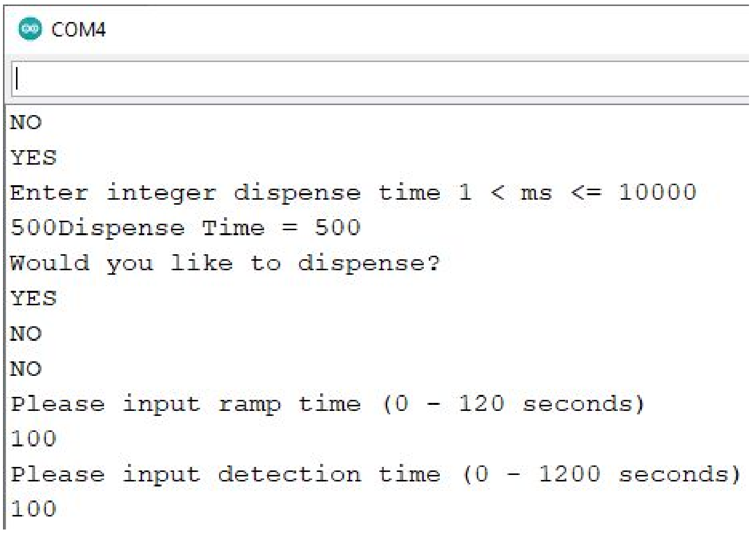

The initial dispense menu has been excited by sending “NO” to “would you like to dispense?”.

The user is prompted to enter a ramp time from 0 to 120 seconds. This is the time allowed between temperature intervals for the heaters to reach and settle at the new temperature

setpoint. Lower temperatures require a shorter amount of time, higher temperatures require a longer amount of time. 100 seconds has been chosen in this instance.

The user will then be prompted to enter a measurement time between 0 and 1200 seconds (20 minutes). This is to accommodate different potentiostats and numbers of electrodes in service. The ramp and detection time setup menu can be reached by running the "END" command during the main process. 100 seconds has been chosen again (arbitrarily).

Ramp Profile Setup:

```
COM4
100
Please input detection time (0 - 1200 seconds)
100
What ramp would you like to use?Please select one of the available ramps by typing the comand that corresponds to the desired ramp
1 C/min = LOW
0.5 C/min = MEDIUM
0.2 C/min = HIGH
LOWramp LOW selected
would you like to change the initial and final temperature of the ramp?
please type YES or NO
Please type the initial temperature like an integer from 25 to 100
Enter the final temp
```

The user can now select a ramp profile from the list of LOW, MED, or HIGH. This alters the temperature interval that will be implemented during the main process. The user can then choose a start and finish temperature for the device to use. These temperatures are not displayed but are taken by the device. In this instance 25 and 50 were entered.

Main Process:

```
COM4
Please type the initial temperature like an integer from 25 to 100
Enter the final temp
Temperature 1:  6.92;Temperature 2:  6.86,en el segundo:  -0.8
25.00 Time = 0 Temperature 1:  4.89;Temperature 2:  4.83,en el segundo:  -1.5
25.00 Time = 0 Temperature 1:  4.89;Temperature 2:  4.82,en el segundo:  -2.3
25.00 Time = 0 Temperature 1:  4.86;Temperature 2:  4.79,en el segundo:  -3.0
25.00 Time = 0 Temperature 1:  4.85;Temperature 2:  4.79,en el segundo:  -3.8
25.00 Time = 0 Temperature 1:  4.85;Temperature 2:  4.78,en el segundo:  -4.6
25.00 Time = 0 Temperature 1:  4.89;Temperature 2:  4.83,en el segundo:  -5.3
25.00 Time = 0 Temperature 1:  4.85;Temperature 2:  4.79,en el segundo:  -6.1
25.00 Time = 0 Temperature 1:  4.83;Temperature 2:  4.77,en el segundo:  -6.8
25.00 Time = 0 Temperature 1:  4.85;Temperature 2:  4.79,en el segundo:  -7.6
0
500ms PUMP
25.00 Time = 0 Temperature 1:  6.51;Temperature 2:  6.45,en el segundo:  -8.4
25.00 Time = 0 Temperature 1:  4.82;Temperature 2:  4.76,en el segundo:  -9.1
25.00 Time = 0
Autoscroll  Show timestamp
```

Once the ramp profile is decided, the main process will start. Here, the current setpoint temperature will be displayed along with "Temperature 1" – the top heater and "Temperature 2" – the bottom heater. Current process time will be displayed as well as "0 seconds".

```
CONTINUING
25.00 Time = 0 Temperature 1:  6.41;Temperature 2:  6.34,en el segundo: -12.9
25.00 Time = 0 Temperature 1:  4.86;Temperature 2:  4.80,en el segundo: -12.9
25.00 Time = 1 Temperature 1:  4.92;Temperature 2:  4.85,en el segundo: -12.9
25.00 Time = 2 Temperature 1:  4.77;Temperature 2:  4.70,en el segundo: -12.9
25.00 Time = 3 Temperature 1:  4.90;Temperature 2:  4.82,en el segundo: -12.9
25.00 Time = 4 Temperature 1:  4.87;Temperature 2:  4.80,en el segundo: -12.9
25.00 Time = 5 Temperature 1:  4.86;Temperature 2:  4.79,en el segundo: -12.9
25.00 Time = 6 Temperature 1:  4.87;Temperature 2:  4.80,en el segundo: -12.9
25.00 Time = 7 Temperature 1:  4.86;Temperature 2:  4.79,en el segundo: -12.9
25.00 Time = 8 Temperature 1:  4.78;Temperature 2:  4.71,en el segundo: -12.9
25.00 Time = 9 Temperature 1:  4.97;Temperature 2:  4.90,en el segundo: -12.9
0
SESSION ENDED
0.00 Time = 0 Please input ramp time (0 - 120 seconds)
Autoscroll  Show timestamp
```

Once Temperature 1 has settled at the starting temperature, sending the "GO" command will begin the ramping process, and "0 seconds" will begin counting to the selected ramp time. The bottom heater supports the top heater and so Temperature 1 is the important reading, Temperature 2 ramps up when the setpoint reaches 40°C.

"END" – Will cause the main process to end and bring the user back to the ramp and detection time setup menu (as shown above).

```
COM4
GO
25.00 Time = 0 Temperature 1:  4.85;Temperature 2:  4.79,en el segundo:  -7.6
0
500ms PUMP
25.00 Time = 0 Temperature 1:  6.51;Temperature 2:  6.45,en el segundo:  -8.4
25.00 Time = 0 Temperature 1:  4.82;Temperature 2:  4.76,en el segundo:  -9.1
25.00 Time = 0 Temperature 1:  4.87;Temperature 2:  4.81,en el segundo:  -9.9
25.00 Time = 0 Temperature 1:  4.81;Temperature 2:  4.75,en el segundo: -10.6
25.00 Time = 0 Temperature 1:  4.83;Temperature 2:  4.77,en el segundo: -11.4
25.00 Time = 0 Temperature 1:  4.74;Temperature 2:  4.68,en el segundo: -12.2
25.00 Time = 0 Temperature 1:  4.85;Temperature 2:  4.78,en el segundo: -12.9
```

"PUMP" – Will run the pump for 0.5 seconds during the main process.

Inside the Box

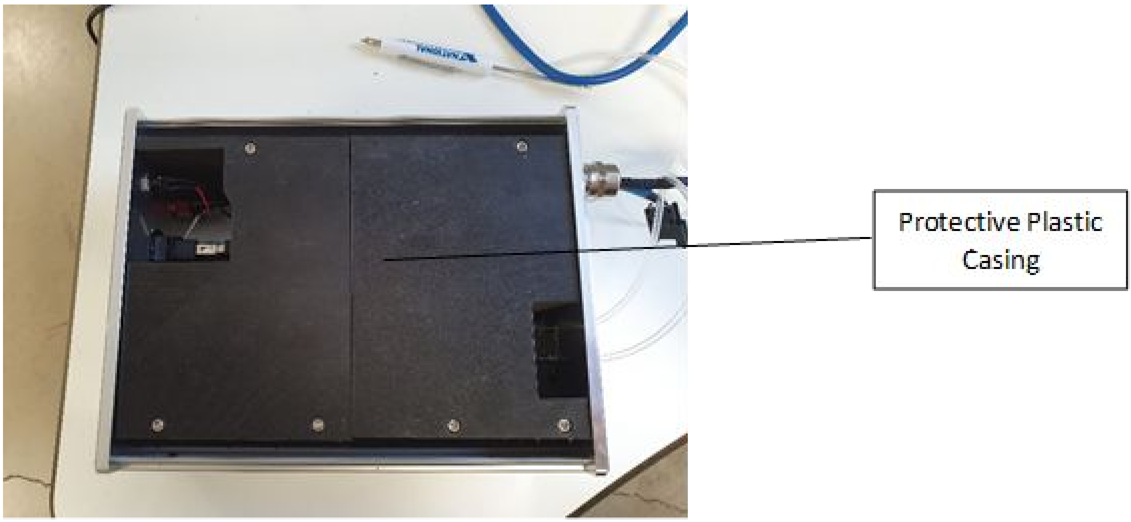

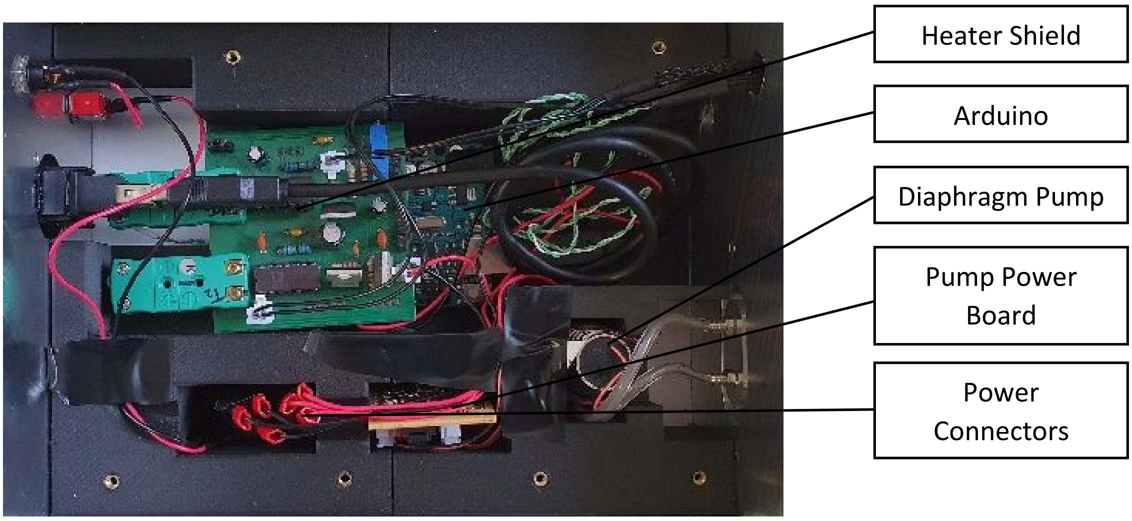

M8 4-way connector wiring (connectors on wires at front of device):
Pin 1 – Heater (No polarity)
Pin 2 – Heater (No polarity)
Pin 3 – Thermocouple -ve
Pin 4 – Thermocouple +ve
